# Associations of distinct sedentary behaviors with cortical, subcortical, and white matter hyperintensity volumes: Evidence from the ARIC study

**DOI:** 10.1002/alz.71582

**Published:** 2026-07-10

**Authors:** Natan Feter, Anamika Nanda, Sarah Hourihan, Daniel Aslan, Jayne Feter, M. Katherine Sayre, Pradyumna K. Bharadwaj, Madeline Ally, Hyun Song, Amit Arora, Silvio Maltagliati, Mark H. C. Lai, Rand R. Wilcox, Yann C. Klimentidis, Gene E. Alexander, David A. Raichlen

**Affiliations:** ^1^ Human and Evolutionary Biology Section, Department of Biological Sciences University of Southern California California USA; ^2^ Postgraduate Program of Epidemiology, School of Medicine Universidade Federal do Rio Grande do Sul Porto Alegre Brazil; ^3^ Department of Human Evolutionary Biology Harvard University Cambridge Massachusetts USA; ^4^ Department of Anthropology University of California Santa Barbara Santa Barbara California USA; ^5^ Department of Psychology University of Arizona Tucson Arizona USA; ^6^ Department of Epidemiology and Biostatistics, Mel and Enid Zuckerman College of Public Health University of Arizona Tucson Arizona USA; ^7^ Laboratoire de Psychologie: Cognition, Comportement, Communication Université Bretagne Sud, LP3C ‐ EA1285 Lorient France; ^8^ Department of Psychology University of Southern California California USA; ^9^ BIO5 Institute University of Arizona Tucson Arizona USA; ^10^ Evelyn F. McKnight Brain Institute University of Arizona Tucson Arizona USA; ^11^ Department of Psychiatry University of Arizona Tucson Arizona USA; ^12^ Neuroscience Graduate Interdisciplinary Program University of Arizona Tucson Arizona USA; ^13^ Physiological Sciences Graduate Interdisciplinary Program University of Arizona Tucson Arizona USA; ^14^ Arizona Alzheimer's Consortium Phoenix Arizona USA; ^15^ Department of Anthropology University of Southern California California USA

**Keywords:** brain structure, dementia, sedentary behavior, white matter hyperintensity

## Abstract

**INTRODUCTION:**

Longitudinal studies linking sedentary behavior (SB) in different contexts to brain structure and white matter hyperintensity (WMH) volume remain limited.

**METHODS:**

We analyzed data from the Atherosclerosis Risk in Communities (ARIC) study (*n* = 1,712). Self‐reported SB was assessed at visit 1 (1987–1989), with neuroimaging (3T magnetic resonance imaging [MRI]) at visit 5 (2011–2013). Participants were non‐demented adults (57% women; 53[5.2] years) who reported frequency of TV watching and occupational sitting. Outcomes included cortical, subcortical, and Alzheimer's disease–signature regions (ADSR), and total WMH brain volumes.

**RESULTS:**

Frequent TV watching was associated with increased WMH volume and reduced frontal, occipital, and ADSR volumes. Sitting during work, which is more cognitively active, was linked to lower WMH and larger frontal (males only), occipital, and parietal volumes. Results remained consistent when adjusted for physical activity.

**DISCUSSION:**

SB is associated with structural brain and WMH volumes. Cognitively active SB may preserve brain structure and cerebrovascular health.

## Highlights

- Sedentary behavior (SB) is associated with structural differences in brain regions vulnerable to Alzheimer's disease, distinct from physical activity.
- The associations between SB and cortical, subcortical, and white matter hyperintensity (WMH) volumes were context‐dependent (e.g., TV watching vs. occupational sitting) and sex‐specific.
- High frequency of TV watching was associated with higher WMH volume and, among males, with reduced cortical and subcortical volumes in regions involved in cognition and emotional processing.
- Occasional sitting during work showed an inverse association with cortical volumes.
- Sitting during work was linked to greater volume of frontal cortices only in males.

## 1 BACKGROUND

Alzheimer's disease (AD) and related dementias (ADRD) represent a growing public health challenge. Although new pharmacological treatments are emerging, behavioral strategies, including those involving lifestyle modification, remain essential for risk reduction. For example, physical activity (PA), especially at moderate to vigorous intensities, has been consistently associated with lower risk of ADRD^1^, ^2^ and numerous studies have linked PA to preserved brain structure in regions implicated in dementia.^3^, ^4^, ^5^, ^6^

Conversely, accumulating evidence suggests that excessive sedentary behavior (SB), such as prolonged sitting, may contribute to elevated ADRD risk.^7^, ^8^ However, fewer studies have examined how SB relates to structural changes in the brain, especially in regions affected early in ADRD progression.^9^, ^10^ Understanding these relationships may be critical, as brain volume and integrity in such regions can serve as early markers of ADRD and can provide insight into the mechanisms through which SB increases risk. Emerging evidence suggests SB may be associated with increased white matter hyperintensity (WMH) volumes,^11^ and decreased regional brain volumes^12^ and cortical thickness of the medial temporal lobe.^13^ However, the link between SB with other brain regions, especially in the “Alzheimer's disease signature regions”, an important imaging biomarker of early neurodegeneration, remains unclear.^14^ The limited evidence is also inconsistent when considering adjustments for PA in the relationships between SB and brain structures.^10^ Some of the observed associations between SB and brain structure persist^12^ while others, including with hippocampal volume and cortical thickness, are attenuated toward the null after accounting for PA.^15^, ^16^

These inconsistencies suggest that examining SB as a single, uniform exposure may obscure important differences. In reality, SB occurs in distinct behavioral contexts that can vary in their cognitive and physiological impact.  For example, TV viewing is often used as a proxy for SB because it is a common sedentary activity, and this cognitively passive SB has been associated with increased dementia risk and lower gray matter volume, even among highly active individuals.^8^, ^13^ However, previous studies suggested that engaging in cognitively active SB, such as video game training or computer use for work, may prevent age‐related brain atrophy and was associated with reduced risk of all‐cause dementia.^8^, ^17^ These findings suggest that the type and context of SB may play a crucial role in determining health outcomes. However, most of the evidence on SB and brain structure comes from cross‐sectional surveys and some prospective studies that fail to differentiate between types of SB.^9^, ^18^

To address these gaps, we examined associations between context‐specific SB and brain structure over a 22‐year follow‐up in non‐demented middle‐aged and older adults from the Atherosclerosis Risk in Communities (ARIC) cohort. We focused on brain volumes known to show early changes in ADRD, including the AD signature regions (a composite of the parahippocampal gyrus, entorhinal cortex, inferior parietal lobule, hippocampus, cuneus, and precuneus regions),^14^ and WMH volume, a marker of small vessel cerebrovascular disease associated with cognitive decline and dementia risk.^19^

## 2 METHODS

### 2.1 Study population

We analyzed data from the ARIC study, an ongoing population‐based prospective study investigating atherosclerotic diseases across four U.S. communities: Washington County, Maryland; Forsyth County, North Carolina; Jackson, Mississippi; and Minneapolis, Minnesota. Detailed information about the ARIC Cohort has been described.^20^ The ARIC study initially enrolled 15,792 adults aged 45 to 64 at visit 1 (1987–1989), with nine follow‐up visits extending until 2023, and neuroimaging was conducted at visit 5 (2011–2013). We included only participants who attended visits 1 and 5 (age range: 67–90 years at visit 5).

**RESEARCH IN CONTEXT**

1. **Systematic review**: Existing evidence supports a link between sedentary behavior (SB), cognitive function, and dementia risk. However, previous systematic reviews have shown that the evidence on the relationship between SB and brain structure or white matter hyperintensity (WMH) volumes is limited and primarily based on cross‐sectional studies that neither account for the context in which SB occurs, nor for participants’ demographics.
2. **Interpretation**: SB is linked with brain structure and WMH volumes, but its effects vary by behavioral context (e.g., TV watching vs. occupational sitting) and sex.
3. **Future directions**: Public health strategies and experimental studies should move beyond total SB time to consider the cognitive demands and context of sedentary activities. Addressing sex‐specific differences may further enhance the impact of public health strategies.

At visit 5 (2011–2013), all surviving members of the original cohort were invited to participate in the ARIC Neurocognitive Study (ARIC‐NCS), an ancillary study designed to investigate determinants of cognitive decline and dementia. As part of this visit, participants underwent an extensive neurocognitive evaluation, and a subset was selected to undergo brain MRI based on standardized eligibility criteria, including the absence of contraindications for MRI and the ability to safely complete the imaging protocol.^21^

**FIGURE 1:**
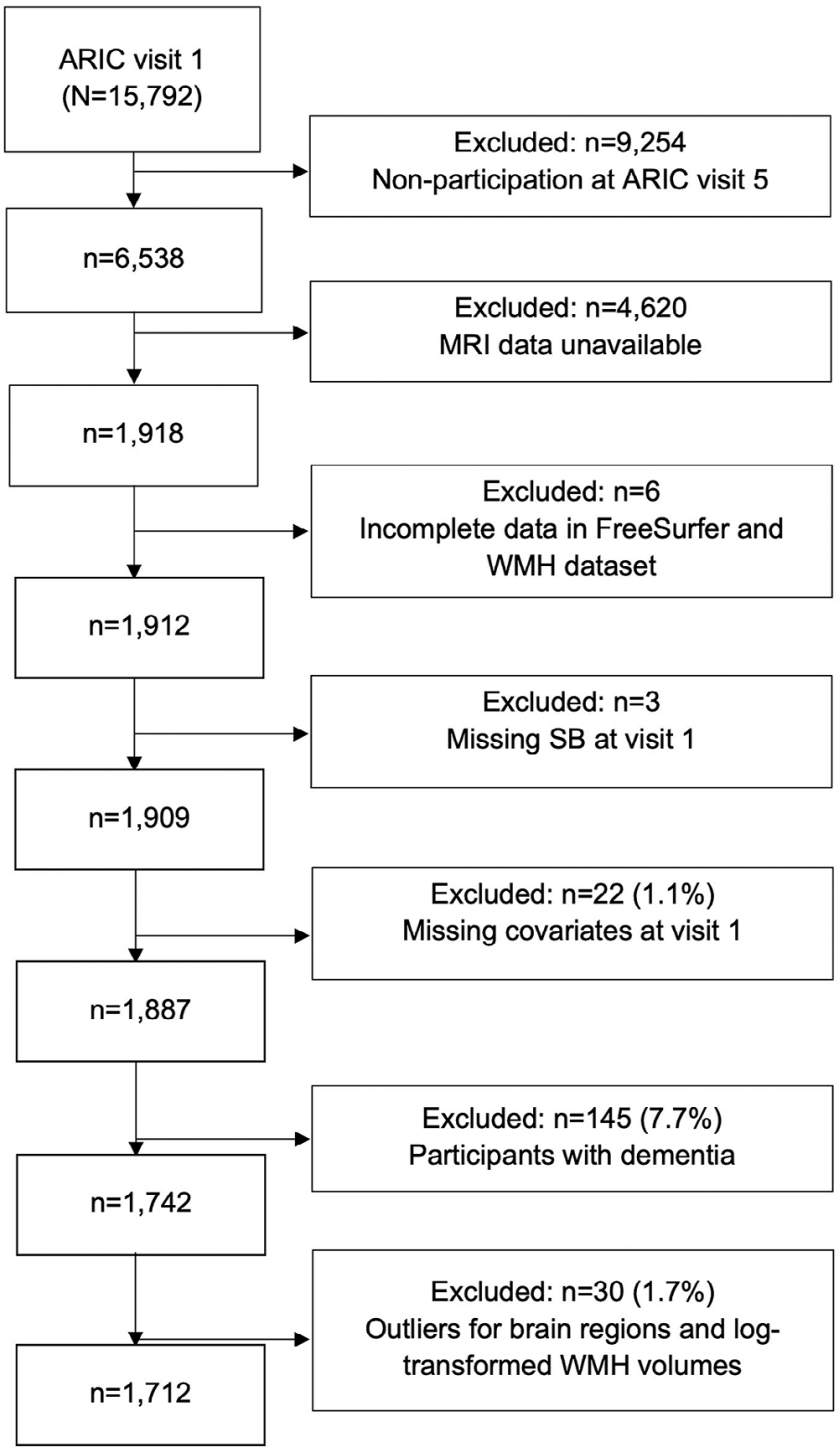
Flow diagram for participants included in the ARIC study study. ARIC, Atherosclerosis Risk in Communities.

We additionally excluded participants who had incomplete magnetic resonance imaging (MRI) data (i.e., poor image quality or missing MRI measures), missing data on self‐reported frequency of TV watching and sitting during work, a diagnosis of dementia defined by an expert‐reviewed cognitive assessment and a computer‐determined syndromic diagnosis at visit 5,^22^ and missing covariates (see Supplementary Methods for more details). Thus, 1,712 participants were included in our analytic sample (Figure 1). We excluded participants with dementia at the time of imaging visit from the main analysis to reduce potential confounding since dementia‐related processes and comorbidities could independently affect brain volume and activity behavior. A full description of the review process for dementia in the ARIC study can be found in the Supplementary Methods 1 and elsewhere.^23^ The institutional review boards of the collaborating universities approved the study protocol, and all participants provided written informed consent.

### 2.2 SB

SB was assessed using the standardized, interviewer‐administered Modified Baecke PA Questionnaire, which measured the frequency of TV watching during leisure time and sitting during work over the 12 months preceding visit 1.^24^ The questionnaire has demonstrated moderate to good reliability (test‐retest reliability ranging from 0.74 to 0.88) and moderate validity (Spearman correlation coefficient 0.54) against energy expenditure measured with doubly labeled water.^24^, ^25^

Participants reported the frequency of TV watching based on the following sentence: “During leisure time do you watch television…”. The response options were “Never”, “Seldom”, “Sometimes”, “Often”, or “Very often”. For sitting during work, the sentence was “At work do you sit…”, with the following options: “Does not work”, “Never”, “Seldom”, “Sometimes”, “Often”, or “Always”.

### 2.3 Brain structure

All brain MRI data were processed and analyzed by the ARIC MRI Reading Center at the Mayo Clinic (Rochester, MN).^26^, ^27^ MRI acquisition protocols were harmonized across study sites using standardized procedures developed by the Mayo Clinic MRI Reading Center. The imaging‐derived variables used in the present study were obtained from the dbGaP repository, where they were made available by ARIC investigators. Brain images were obtained at visit 5 using 3T MRI scanners manufactured by Siemens (Erlangen, Germany), including the Magnetom Skyra (North Carolina and Mississippi study centers), Magnetom Trio (Minnesota study center), and Magnetom Verio (Maryland study center). ARIC‐NCS brain MRI acquisition used site‐specific configurations as follows: Forsyth County, Magnetom Skyra with a 32‐channel head coil; Jackson, Magnetom Skyra with a 20‐channel head coil; Minneapolis, Magnetom Trio with a 12‐channel head coil; and Washington County, Magnetom Verio with a 12‐channel head coil.^28^ Volumes of regions of interest (ROI), reported in cm^3^, were obtained from T1‐weighted scans using FreeSurfer image analysis software (version 5.1; Laboratory for Computational Neuroimaging).^29^ All images were processed centrally using standardized pipelines, minimizing inter‐scanner variability in derived measures.

Although multiple sequences were acquired as part of the ARIC protocol, the present analyses used scan measures derived from the following sequences: magnetization‐prepared rapid gradient echo (MPRAGE) (1.2‐mm slices) and axial T2 fluid‐attenuated inversion recovery (FLAIR) (5‐mm slices). Brain volumes were measured on MPRAGE sequences using image analysis software (FreeSurfer; http://surfer.nmr.mgh.harvard.edu).^27^, ^29^ The Mayo Aging and Dementia Imaging Research (ADIR) Lab developed and distributed the protocol for each scanner in the study. Detailed MRI acquisition and quality control procedures are described in ARIC Neurocognitive Study Manual 13 and related publications.^27^, ^30^, ^31^

WMH volumes were measured using a semiautomated segmentation algorithm on T2‐weighted FLAIR images.^32^, ^33^, ^34^ The MPRAGE image was resampled to the FLAIR image space to minimize false‐positive WMH detections derived from FLAIR. White matter masks were subsequently generated from tissue segmentation of the MPRAGE images. White matter hyperintensities were then identified using an automated, slice‐wise approach that combined seed initialization with a region‐growing algorithm.^32^, ^33^, ^34^, ^35^

The Mayo ADIR Lab reviewed all scans for medically significant abnormalities. A trained image analyst evaluated all scans for protocol compliance and scan quality. Quality control data were entered into data forms and transmitted to the coordinating center. Quality problems with any scan resulted in a request for a rescan. Full details of image processing methods have been published ^27^, ^31^, ^35^, ^36^, ^37^, ^38^ and are available online, including imaging and quality control protocols.^30^, ^39^, ^40^ We included study site as a covariate in all statistical models to further account for potential between‐site variability.

We used the *ggseg* package in R (version 4.4.3)^41^ to merge the findings from generalized linear models in Desikan–Killiany and subcortical segmentation atlases to visualize the potential association between 80 regions of interest (ROIs) (68 ROIs from Desikan‐Killiany atlas and 12 from subcortical segmentation atlas) and SB. A full list of ROIs is available in Supplementary Methods 2. We also used pre‐defined regionally combined volumetric variables provided in the ARIC neuroimaging dataset. These included measures of brain structure volumes grouped across hemispheres: lobar volumes (frontal, temporal, occipital, and parietal), subcortical deep gray matter volume (combined volume of the thalamus, caudate, putamen, and pallidum), and the AD signature region volume (combined volume of the parahippocampal gyrus, entorhinal cortex, inferior parietal lobule, hippocampus, cuneus, and precuneus).^27^, ^42^ Due to the skewed nature of WMH volumes, values were log‐transformed for analysis. Total intracranial volume (TIV) was estimated from Freesurfer.^43^ Finally, we checked structural MRI scan values for outliers. We additionally excluded 26 participants with values three standard deviations above the sample mean in any of the following neuroimaging outcomes: frontal, temporal, occipital, and parietal lobes volumes, the log‐transformed WMH volume, and deep gray subcortical structure volume.

### 2.4 Covariates

Covariates included age at visit 5 (years), sex (female, male), education level (less than high school, high school or equivalent or greater), and occupational status (managerial and professional specialty occupations, technical, sales, and administrative support occupations, service occupations, farming, forestry, and fishing occupations, precision production, craft, and repair occupations, operators, fabricators, and laborers, homemakers, retired, other). Due to the sampling strategies, we developed race‐center as a five‐level categorical variable combining race and field center (Minnesota /White, Maryland/White, North Carolina/White, North Carolina/Black, Mississippi/Black).^44^, ^45^ We also included smoking status (never, former, current), drinking status (never, former, current), body mass index (BMI), PA, hypertension (yes, no), and diabetes (yes, no) at visit 1. BMI was calculated using weight and height assessed during clinic visit at visit 1.

PA was also assessed using the modified Baecke PA Questionnaire at ARIC at visits 1 and 5. Participants reported the frequency of sports during leisure time over the past year. The summary sports index was estimated by multiplying the weekly frequency (i.e., never, seldom, sometimes, often, very often) of each sports activity (up to four) by the duration (i.e., hours per week) and the proportion of the year participants practiced such sports activity (i.e., number of months in the last year). The summary sports index ranges from 1 to 5 (with higher score reflecting greater activity levels).^24^

Hypertension was defined as systolic blood pressure ≥140 mmHg, diastolic blood pressure ≥90 mmHg, or using blood pressure‐lowering medication. Diabetes mellitus was defined as fasting glucose ≥126 mg/dL, non‐fasting glucose ≥200 mg/dL, self‐reported physician‐diagnosed diabetes, or use of diabetes medication. All analyses were also adjusted for TIV at visit 5 to account for differences in head size among participants.

### 2.5 Statistical analysis

Categorical variables were reported as frequencies and percentages, while continuous variables were presented as means and standard deviations or as medians and interquartile ranges (IQR), depending on data normality, assessed via histograms and skewness coefficients.

Multivariate generalized linear models examined associations between TV watching (Never/Seldom [reference category for analyses], Sometimes, Often, Very often) and sitting during work (Does not work, Never/Seldom [reference category for analyses], Sometimes, Often, Always) with regional brain volumes and total WMH volume. Four sets of models were considered: the crude model, adjusted only for estimated TIV at visit 5; the minimally adjusted model, additionally adjusted for sex, age at visit 5, race‐center, education, and occupation; the adjusted model, further adjusted for smoking, drinking, BMI, diabetes, and hypertension at visit 1, and mutually adjusted for frequency of TV watching and sitting during work; and the fully adjusted model, additionally adjusted for PA at visit 1. False discovery rate (FDR) control was implemented using the Benjamini–Hochberg procedure to adjust *p*‐values for multiple comparisons. In the ROI‐wise analysis, FDR correction was applied across all 80 Freesurfer‐derived regional brain volumes, producing a single set of adjusted *p*‐values for the association between SB and each ROI. For the lobar and composite MRI outcomes, we extracted the *p*‐values for all SB levels across all grouped volumes and applied the Benjamini–Hochberg procedure across all of these comparisons.^46^ We conducted sex‐stratified analyses due to the known associations between sex and SB.^47^, ^48^

We also performed sensitivity analyses. First, we included apolipoprotein E (*APOE)* ɛ4 genotype (0 or ≥1 ε4 allele) as a covariate. We conducted a sensitivity analysis including the APOE ε4 genotype because genotype data were unavailable for 890 participants. In this analysis, APOE ε4 was entered as a covariate given its well‐established association with adverse brain outcomes, particularly dementia, as well as structural brain differences and white matter pathology in cognitively normal adults.^49^, ^50^, ^51^, ^52^ Second, we excluded 676 participants with mild cognitive impairment (MCI) and 100 identified with cerebrovascular disease‐related MCI at visit 5 to further minimize the influence of early cognitive changes on neuroimaging outcomes. Cerebrovascular disease‐related MCI was adjudicated by one or two experts based on the history of stroke with abrupt cognitive decline, evidence of bilateral or multiple infarcts or extensive WMH on imaging, and physical examination findings consistent with a typical stroke pattern. MCI was determined using a predefined algorithm incorporating cognitive performance across multiple domains, longitudinal cognitive change, and functional status measures (see Supplementary Methods for full details). Third, we used inverse probability of attrition weighting to investigate the potential impact of selective loss to follow‐up. We estimated each participant's probability of attending visit 5 using a logistic regression model including baseline characteristics potentially associated with attrition and the exposures of interest: age, sex, race‐center, education, occupation, smoking status, alcohol use, PA, BMI, hypertension, diabetes, and SB. Stabilized weights were calculated as the mean probability of follow‐up divided by the individual's predicted probability of follow‐up. To minimize the influence of extreme values, weights were truncated at the 1st and 99th percentiles. These weights were combined with the visit 5 sampling weights to account for the stratified random sampling used to select participants for MRI. Fourth, we performed generalized linear models using time‐varying covariates derived from repeated assessments across visits 1 (1987–1989), 3 (1993–1995), and 5 (2011–2013): smoking status, alcohol use, PA, BMI, hypertension, and diabetes. Finally, we included context‐specific SB as a time‐varying exposure in the time‐varying models. Because TV watching and sitting at work were assessed concurrently only in visits 1 and 3, we did not explore the time‐dependent association across the entire follow‐up.

All analyses incorporated sampling weights to account for the stratified random sampling approach used to select persons to receive an MRI to reflect the sample of ARIC participants who attended visit 5. Analyses were performed using R (version 4.4.3, R Foundation for Statistical Computing, Vienna, Austria).

## 3 RESULTS

### 3.1 Participants characteristics

Table 1 presents the sociodemographic, behavioral, and clinical characteristics of the 1,712 participants included in the study. Participants had a mean age of 76 years (SD: 5.2) at visit 5 and were more than half female (57.3%). The median follow‐up period was 23.7 (IQR: 23.1, 25.1) years. More than half (61%) were current drinkers, and 19.5% were current smokers. Diabetes was more prevalent among men, who also showed a higher frequency of sitting during work compared to women (often: 35% vs. 19%, respectively; Table S1). We observed that 11.4% of participants reported both often or very often TV viewing and often or always sitting at work.

**TABLE 1:** Characteristics of the participants included in the ARIC study.

Parameter	Female (*N* = 981)	Male (*N* = 731)	Overall (*N* = 1712)
Age at visit 1 in years, mean (SD)	53 (5.1)	53 (5.3)	53 (5.2)
Age at visit 5 in years, mean (SD)	76 (5.2)	77 (5.3)	76 (5.2)
Schooling (*< high school education)*, *n* (%)	163 (16.6%)	88 (12.0%)	251 (14.7%)
Study center—race, *n* (%)			
Minnesota—White	215 (21.9%)	221 (30.2%)	436 (25.5%)
Maryland—White	299 (30.5%)	176 (24.1%)	475 (27.7%)
North Carolina—White	215 (21.9%)	179 (24.5%)	394 (23.0%)
North Carolina—Black	12 (1.2%)	10 (1.4%)	22 (1.3%)
Mississippi—Black	240 (24.5%)	145 (19.8%)	385 (22.5%)
Occupation, *n* (%)			
Managerial and professional specialty	203 (20.7%)	282 (38.6%)	485 (28.3%)
Technical, sales, and administrative support	295 (30.1%)	134 (18.3%)	429 (25.1%)
Service occupations	151 (15.4%)	41 (5.6%)	192 (11.2%)
Farming, forestry, and fishing	0 (0%)	5 (0.7%)	5 (0.3%)
Precision production, craft, and repair	31 (3.2%)	92 (12.6%)	123 (7.2%)
Operators, fabricators, and laborers	56 (5.7%)	83 (11.4%)	139 (8.1%)
Homemakers	150 (15.3%)	0 (0%)	150 (8.8%)
Retired	67 (6.8%)	94 (12.9%)	161 (9.4%)
Other	28 (2.9%)	0 (0%)	28 (1.6%)
Current smoking, *n* (%)			
Current smoker	201 (20.5%)	132 (18.1%)	333 (19.5%)
Former smoker	212 (21.6%)	340 (46.5%)	552 (32.2%)
Never smoker	568 (57.9%)	259 (35.4%)	827 (48.3%)
Alcohol consumption, *n* (%)			
Current drinker	543 (55.4%)	505 (69.1%)	1048 (61.2%)
Former drinker	130 (13.3%)	104 (14.2%)	234 (13.7%)
Never drinker	308 (31.4%)	113 (15.5%)	421 (24.6%)
Physical activity score, mean (SD)	2.4 (0.76)	2.7 (0.87)	2.5 (0.82)
Body mass index, *n* (%)	27 (5.4)	27 (3.3)	27 (4.7)
Underweight	8 (0.8%)	1 (0.1%)	9 (0.5%)
Normal weight	387 (39.4%)	199 (27.2%)	586 (34.2%)
Overweight	289 (29.5%)	409 (56.0%)	698 (40.8%)
Obese	297 (30.3%)	122 (16.7%)	419 (24.5%)
Hypertension (yes), *n* (%)	262 (26.7%)	160 (21.9%)	422 (24.6%)
Diabetes (yes), *n* (%)	53 (5.4%)	47 (6.4%)	100 (5.8%)
TV watching, *n* (%)			
Never/seldom	201 (20.5%)	109 (14.9%)	310 (18.1%)
Sometimes	455 (46.4%)	369 (50.5%)	824 (48.1%)
Often	268 (27.3%)	215 (29.4%)	483 (28.2%)
*Very often*	57 (5.8%)	38 (5.2%)	95 (5.5%)
Sitting during work, *n* (%)			
Never/seldom	265 (27.0%)	152 (20.8%)	417 (24.4%)
Sometimes	209 (21.3%)	203 (27.8%)	412 (24.1%)
Often	185 (18.9%)	253 (34.6%)	438 (25.6%)
Always	136 (13.9%)	67 (9.2%)	203 (11.9%)
Unemployed	186 (19.0%)	56 (7.7%)	242 (14.1%)

*Note*: All variables were assessed at visit 1. Age was also assessed at visit 5. *N* = 1712.

Abbreviations: ARIC, Atherosclerosis Risk in Communities; SD, standard deviation.

### 3.2 Context‐specific association between SB and cortical and subcortical volume

**FIGURE 2:**
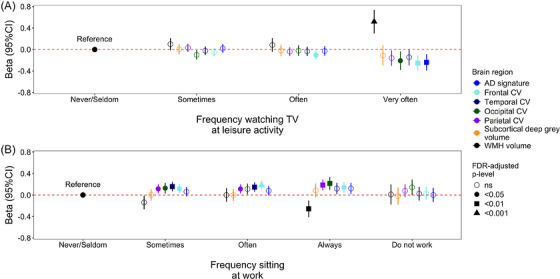
Association between brain structure and the frequency of watching TV in leisure time (A) and sitting during work (B). *N* = 1,712. ARIC study. Generalized linear models adjusted for sex, race‐center, occupational status, schooling, body mass index, diabetes, hypertension, physical activity at visit 1, age and total intracranial volume at visit 5, and the frequency of TV watching and sitting during work. AD signature region includes volume of the parahippocampal, entorhinal, inferior parietal lobules, hippocampus, and precuneus. The figure illustrates brain regions that remained associated with sedentary behavior after adjustment for multiple comparisons (A, 21 comparisons; B, 28 comparisons). AD, Alzheimer disease; ARIC, Atherosclerosis Risk in Communities; CI, confidence interval; CV, cortical volume; WMH, white matter hyperintensity.

We found a context‐dependent association between SB and several grouped brain regions. As shown in Figure 2, watching TV very often was significantly associated with a lower volume in the frontal (−0.25; 95% confidence interval [CI]: −0.40, −0.11), occipital (−0.21; 95% CI: −0.38, −0.04), and in AD signature (beta: −0.24; 95% CI: −0.39, −0.09) regions, and with higher WMH volume (0.52; 95% CI: 0.30, 0.74) (Table S2). In contrast, participants who reported always sitting during work showed a significantly lower WMH volume (−0.26; 95% CI: −0.41, −0.10). In addition, these participants showed higher parietal (0.19; 95% CI: 0.08, 0.29), occipital (0.22; 95% CI: 0.09, 0.34), and frontal (0.14; 95% CI: 0.04, 0.24) cortical volumes than those who reported never or seldom (Figure 2). All associations were stronger in models adjusting only for TIV (crude model) compared to minimally adjusted models, and the strength of the associations did not substantially change with further adjustments.

### 3.3 Associations between SB and brain structure after adjusting for PA

Including PA at visits 1 and 5 in the model also did not yield meaningful association changes (Tables S2 and S3). Adjusting for PA at visit 5, greater TV watching remained associated with lower volume in the AD signature (−0.21; 95% CI: −0.36, −0.06), occipital (−0.21; 95% CI: −0.39, −0.04), and frontal regions (−0.24; 95% CI: −0.39, −0.10), and higher WMH volume (0.48; 95% CI: 0.26, 0.69). When adjusted for PA at visit 5, compared with never or seldom sitting, high levels of sitting during work (i.e., always) were associated with higher frontal (0.13; 95% CI: 0.03, 0.23), occipital (0.20; 95% CI: 0.08, 0.32), and parietal (0.17; 95% CI: 0.07, 0.27) cortical volumes. Less frequent sitting (sometimes) was additionally associated with higher frontal and temporal cortical volumes. These findings suggest that the context‐dependent association between SB and brain structure is at least partially independent of PA and other behavioral and cardiometabolic risk factors.

### 3.4 SB and specific brain regions

**FIGURE 3:**
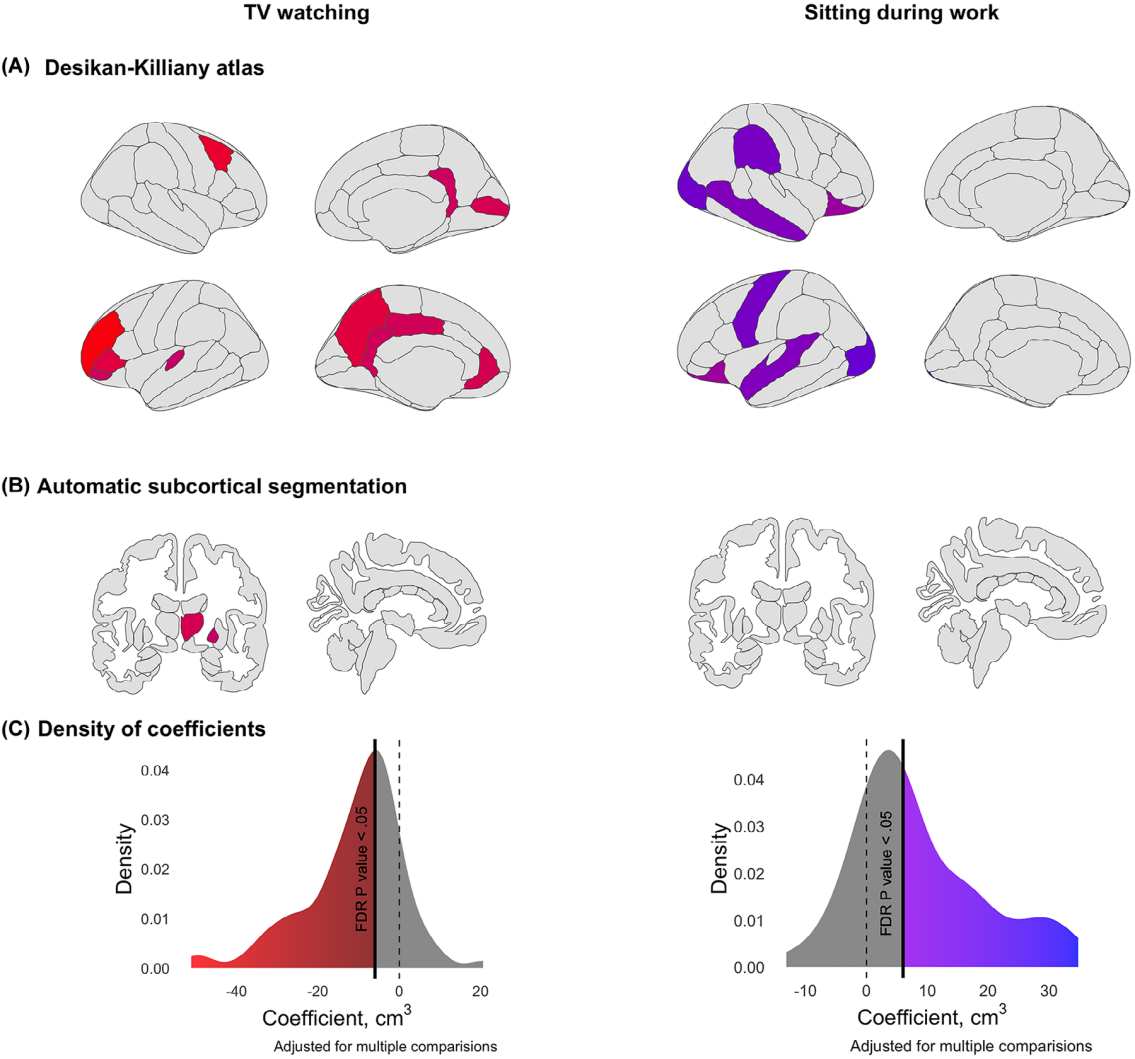
Brain regions associated with the frequency of TV watching (left) and sitting during work (right). *N* = 1,712. ARIC study. (A) Lateral (first column) and medial (second column) views of the right (top row) and left (bottom row) cerebral hemispheres. (B) The coronal and sagittal sides. Red areas in A, B, and C indicate that watching TV very often during leisure time was associated with a lower cortical volume compared to the reference category: “Never/seldom”. Blue areas in A, B, and C indicate that always sitting during work was associated with a higher cortical volume compared to the reference category: “Never/seldom”. (A) Sitting during work, a small colored region in the frontal cortex (frontal pole) shows higher cortical volume in participants who reported always sitting during work than those who reported never or seldom sitting. Generalized linear models included sex, race‐center, occupational status, schooling, body mass index, diabetes, hypertension, physical activity at visit 1, age and total intracranial volume at visit 5, and the frequency of TV watching and sitting during work. The figure illustrates the 80 regions of interest identified using Desikan–Killiany and subcortical segmentation atlases that remained associated with high sedentary behavior (i.e., A, very often TV watching; B, always sitting at work) after controlling for multiple comparisons using the FDR procedure. ARIC, Atherosclerosis Risk in Communities; FDR, false discovery rate.

Figure 3 illustrates the brain regions with a statistically significant difference in volume between the highest frequency of TV watching (Very often) and sitting during work (Always) and the lowest (Never/Seldom) after adjustment for multiple comparisons. We found that TV watching was associated (FDR *p*‐value ≤ 0.047) with lower volume in specific brain regions in the right hemisphere (pericalcarine cortex, caudal middle frontal gyrus, superior frontal gyrus, isthmus cingulate, thalamus proper, pallidum) and left hemisphere (pars orbitalis, pars triangularis, rostral anterior cingulate cortex, rostral middle frontal gyrus, isthmus cingulate, posterior cingulate cortex, precuneus, transverse temporal gyrus) (Table S4). In contrast, prolonged sitting during work was associated (FDR *p*‐value ≤ 0.049) with higher volume in regions of the right hemisphere (frontal pole, lateral orbitofrontal cortex, lateral occipital cortex, supramarginal gyrus, superior parietal lobule, and middle temporal gyrus) and the left hemisphere (lateral occipital cortex, lateral orbitofrontal cortex, superior temporal gyrus, and precentral gyrus) (see Table S5).

### 3.5 Sex‐specific association between SB and brain structure

We performed sex‐stratified analyses based on significant interaction terms between sex and context‐specific SB (Tables S6−S9). In women, no brain regions were linked to very often TV watching and sitting during work (Figure S1). In contrast, in men, a moderate and high frequency of TV watching was linked (FDR *p*‐value ≤ 0.021) to lower volume in frontal, temporal, occipital, parietal, subcortical deep gray structures, and AD signature regions (Figure S2). In addition, a moderate frequency was associated with lower volume in occipital (FDR *p*‐value = 0.009) and temporal (FDR *p*‐value = 0.030) regions.

In men, a high frequency of TV watching was also associated (FDR *p*‐value ≤ 0.037) with reduced cortical and subcortical volume across multiple brain regions in both hemispheres (Figure S3). These included frontal regions (i.e., bilateral rostral middle frontal gyrus, left pars triangularis, bilateral pars orbitalis, right caudal middle frontal gyrus, and right superior frontal gyrus), parietal regions (i.e., bilateral inferior parietal lobule, left supramarginal gyrus, left precuneus, and right precuneus), temporal regions (i.e., right temporal pole, right middle temporal gyrus, right inferior temporal gyrus, left parahippocampal gyrus, and left superior temporal gyrus), and limbic and subcortical regions (i.e., left rostral anterior cingulate cortex, left insula, bilateral isthmus of the cingulate cortex, right posterior cingulate cortex, bilateral putamen, and bilateral pallidum).

In contrast, always sitting during work was associated (FDR *p*‐value ≤ 0.002) with higher volume in the right lateral orbitofrontal cortex, left lateral occipital cortex, right frontal pole, left paracentral lobule, left isthmus of the cingulate cortex, right precuneus, and right superior frontal gyrus. In women, always sitting during work was linked to lower rostral anterior cingulate cortex in the left hemisphere (FDR *p*‐value = 0.041). No other brain regions were significantly associated with high levels of TV watching or occupational sitting in women.

### 3.6 Sensitivity analyses

Frequent TV watching remained associated with a lower cortical volume in AD signature regions (−0.19; 95% CI: −0.35, −0.03; FDR *p*‐value = 0.043) and increased WMH volume (0.42; 95% CI: 0.19, 0.66; FDR *p*‐value = 0.003) after excluding 100 participants with cerebrovascular disease‐related MCI at visit 5 (Table S10). Additionally, frequent sitting during work was linked to higher cortical volume in the AD signature (0.15; 95% CI: 0.04, 0.26; FDR *p*‐value = 0.018), as well as in frontal (0.13; 95% CI: 0.03, 0.23; FDR *p*‐value = 0.038), occipital (0.23; 95% CI: 0.10, 0.35; FDR *p*‐value = 0.002), and parietal (0.20; 95% CI: 0.09, 0.30; FDR *p*‐value = 0.001) regions. Moderate sitting during work was also associated with higher volume in the temporal region (0.14; 95% CI: 0.05, 0.23; FDR *p*‐value = 0.010). Similar results were seen after excluding 676 participants with MCI; during the visit, always sitting during work was associated with lower WMH volume (−0.27; 95% CI: −0.46, −0.08; FDR *p*‐value = 0.015) and higher cortical volume in the AD signature regions (0.16; 95% CI: 0.03, 0.30; FDR *p*‐value = 0.048), as well as in frontal (0.17; 95% CI: 0.04, 0.30; FDR *p*‐value = 0.038), occipital (0.21; 95% CI: 0.06, 0.36; FDR *p*‐value = 0.002), and parietal (0.22; 95% CI: 0.09, 0.35; FDR *p*‐value = 0.001) regions. Moderate sitting during work was also associated with higher volume in the temporal region (0.15; 95% CI: 0.04, 0.26; FDR *p*‐value = 0.010). However, the association between TV watching and the volume in the AD signature regions did not remain statistically significant after FDR adjustment, though the direction of effect remained (−0.21; 95% CI: −0.40, −0.02; FDR *p*‐value = 0.080) (Table S11).

Most of the associations with TV watching remained after adjusting for the *APOE* ɛ4 genotype (Table S12). Specifically, frequent TV watching was associated with higher WMH volume (0.62; 95% CI: 0.23, 1.00; FDR *p*‐value = 0.007) and reduced volume in the frontal (−0.37; 95% CI: −0.62, −0.11; FDR *p*‐value = 0.016) and occipital (−0.47; 95% CI: −0.79, −0.15; FDR *p*‐value = 0.012) regions. Conversely, always sitting during work was only linked to lower WMH volume (−0.30; 95% CI: −0.54, −0.06; FDR *p*‐value = 0.047). Results were materially unchanged in the analysis using inverse probability weighting to address potential attrition bias (Table S13). Frequent TV watching remained associated with reduced volume in the frontal (−0.20; 95% CI: −0.33, −0.07; FDR *p*‐value = 0.012) and higher WMH volume (0.49; 95% CI: 0.29, 0.68; FDR *p*‐value < 0.001). However, the association with AD signature regions did not remain statistically significant after FDR adjustment, though the direction of effect remained (−0.15; 95% CI: −0.29, −0.02; FDR *p*‐value = 0.057). Frequent sitting during work was linked to lower WMH volume (−0.24; 95% CI: −0.40, −0.09; FDR *p*‐value = 0.008) and higher cortical volume in the AD signature (0.14; 95% CI: 0.03, 0.24; FDR *p*‐value = 0.035), as well as in frontal (0.12; 95% CI: 0.02, 0.23; FDR *p*‐value = 0.039), occipital (0.29; 95% CI: 0.17, 0.41; FDR *p*‐value < 0.001), and parietal (often: 0.11; 95% CI: 0.02, 0.19; FDR *p*‐value = 0.034; always: 0.16; 95% CI: 0.06, 0.26; FDR *p*‐value = 0.008) regions. Moderate sitting during work was also associated with higher volume in the temporal region (0.12; 95% CI: 0.03, 0.21; FDR *p*‐value = 0.025).

Analysis using time‐varying covariates yielded similar results (Table S14). Frequent TV watching was associated with lower volume in AD signature (−0.21; 95% CI: −0.36, −0.06; FDR *p*‐value = 0.017) and frontal (−0.23; 95% CI: −0.38, −0.09; FDR *p*‐value = 0.004) regions and higher WMH volume (0.43; 95% CI: 0.21, 0.64; FDR *p*‐value < 0.001). Occasional (i.e., sometimes) sitting at work was associated with higher volume in frontal (0.10; 95% CI: 0.02, 0.18; FDR *p*‐value = 0.047), temporal (0.14; 95% CI: 0.05, 0.22; FDR *p*‐value = 0.006) and occipital (0.12; 95% CI: 0.02, 0.22; FDR *p*‐value = 0.042) regions. Frequent sitting at work was associated with higher volumes in frontal (often: 0.18; 95% CI: 0.09, 0.26; FDR *p*‐value < 0.001), temporal (often: 0.14; 95% CI: 0.06, 0.23; FDR *p*‐value = 0.005), occipital (always: 0.20; 95% CI: 0.08, 0.33; FDR *p*‐value = 0.004), parietal (often: 0.11; 95% CI: 0.03, 0.19; FDR *p*‐value = 0.021; always: 0.14; 95% CI: 0.04, 0.24; FDR *p*‐value = 0.013) regions and lower WMH volume (always: −0.24; 95% CI: 0.40, 0.09; FDR *p*‐value = 0.011).

Findings were also consistent when context‐specific SB was used as a time‐varying exposure (Table S15). Frequent TV watching was associated with lower volume in the AD signature (−0.21; 95% CI: −0.36, −0.06; FDR *p*‐value = 0.019) and frontal (often: −0.10; 95% CI: −0.18, −0.01; FDR *p*‐value = 0.049; very often: −0.23; 95% CI: −0.38, −0.09; FDR *p*‐value = 0.004) regions, and higher WMH volume (0.42; 95% CI: 0.21, 0.64; FDR *p*‐value < 0.001). In contrast, occasional sitting at work was associated with higher volume in frontal (0.10; 95% CI: 0.02, 0.18; FDR *p*‐value = 0.044), temporal (0.14; 95% CI: 0.05, 0.22; FDR *p*‐value = 0.007), and occipital (0.12; 95% CI: 0.02, 0.22; FDR *p*‐value = 0.040) regions. Frequent sitting at work was associated with higher volumes in frontal (often: 0.18; 95% CI: 0.09, 0.26; FDR *p*‐value < 0.001), temporal (often: 0.14; 95% CI: 0.06, 0.23; FDR *p*‐value = 0.005), occipital (often: 0.12; 95% CI: 0.02, 0.22; FDR *p*‐value = 0.047; always: 0.21; 95% CI: 0.09, 0.33; FDR *p*‐value = 0.003), and parietal (often: 0.11; 95% CI: 0.03, 0.19; FDR *p*‐value = 0.021; always: 0.14; 95% CI: 0.04, 0.24; FDR *p*‐value = 0.014) regions and lower WMH volumes (always: −0.24; 95% CI: −0.39, −0.08; FDR *p*‐value = 0.008).

## 4 DISCUSSION

This study investigated the association between SB in different contexts and brain structures in a large sample of non‐demented older adults, offering novel insights into region‐specific, context‐dependent, and sex‐specific associations. Our findings indicate that frequent TV watching was associated with adverse brain structural outcomes, including lower cortical volume in AD‐related regions and greater WMH lesion volume. In contrast, sitting during work was associated with higher cortical volumes in selected brain regions. Most of these associations persisted after multiple sensitivity analyses excluding participants with cerebrovascular disease and those with MCI and adjusting for several sociodemographic, behavioral, and clinical variables. Associations with TV watching were also maintained after adjusting for *APOE* ɛ4 genotype. These findings suggest robust links between SB context and brain health.

### 4.1 Context‐dependent associations with SB

Our findings underscore the importance of SB context in its relationship with brain structure. While frequent TV watching was consistently associated with lower cortical volume and greater WMH volume, sitting during work was linked to higher cortical volumes in several regions, including those often considered important for higher‐level executive functions.^53^ The associations with frequent SB weakened when accounting for PA at visit 5, suggesting a potential nonlinear or threshold effect. In sensitivity analyses investigating attrition bias, associations with frontal lobe volume and WMH remained materially unchanged. The association with AD signature region volume was attenuated and did not reach statistical significance after FDR correction, though the direction of effect was preserved, suggesting that selective attrition may have modestly influenced the magnitude of this particular association.

Occasional SB at work may reflect cognitively engaging sedentary tasks, such as reading or problem‐solving, which may potentially confer neuroprotective effects. However, prolonged sitting time may lead to deleterious physiological consequences such as reduced cerebral blood flow and neural efficiency,^54^, ^55^ even during cognitively demanding sedentary activities. This hypothesis aligns with evidence that breaking up extended sedentary periods improves cognitive performance, especially in executive functions.^56^ Future research should examine whether the cognitive benefits of task‐related engagement can offset the physiological risks of sustained SB and whether optimal patterns of engagement and movement can be identified in occupational settings.

Our findings on the context‐dependent association between SB and brain structure are aligned with previous evidence regarding cognitive outcomes. For example, more time spent watching TV was associated with poor cognitive performance, rapid cognitive decline, and a higher risk of dementia in middle‐aged and older adults from the UK Biobank study.^8^, ^57^ In contrast, more time spent using computers showed inverse associations with cognitive function and dementia risk.^8^, ^57^ Similarly, a cross‐sectional analysis of data from middle‐aged and older adults in the Netherlands showed that occupational SB was linked to higher global cognitive function while no association was observed with leisure SB, corroborating the context‐dependent association between SB and cognitive outcomes.^58^ Combined, this emerging evidence suggests that not all sedentary time is equal for brain health;^8^, ^9^ its impact may depend on the nature of the activity and cognitive engagement. This finding is particularly relevant for aging populations, where leisure time comprises a significant portion of daily activity.^59^

### 4.2 WMH and cerebrovascular burden

Our findings also indicate that TV watching, but not sitting during work, is a key predictor of WMH volume, a marker of small vessel cerebrovascular disease and a known predictor of cognitive decline.^60^, ^61^ Importantly, this association remained significant in sensitivity analyses excluding participants with a history of cerebrovascular disease. Elevated WMH volume may reflect cumulative vascular risk associated with prolonged SB, potentially mediated by impaired cerebral perfusion, metabolic dysregulation, or inflammation.^62^, ^63^, ^64^ Although a previous systematic review reported mixed evidence regarding the relationship between SB and WMH volume, the included studies did not consider the context‐specific nature of SB.^65^ Moreover, the existing evidence is largely based on cross‐sectional data, underscoring the novelty of our findings.^65^, ^66^ A separate analysis using UK Biobank data examined device‐measured PA as a moderator in the link between SB and WMH volume relationship.^11^ The authors found that adjusting for PA did not substantially alter the association, aligning with our results, although the link was stronger among individuals with lower PA levels. These findings highlight the potential for SB to independently contribute to cerebrovascular burden.

### 4.3 Sex‐specific differences

Sex‐specific analyses revealed notable differences in the associations between SB and some aspects of brain structure. While not all brain outcomes linked to SB showed interactions with sex, the widespread associations observed in males suggest that they may be more vulnerable to the negative physiologic effects of SB.^67^, ^68^ While our results cannot identify mechanisms driving these differences, it is possible that differences in hormone levels or lifestyle factors such as the type or intensity of leisure activities may play a role in sex‐based variation in outcomes.^67^, ^69^ It is also possible that reporting biases or differing engagement patterns in cognitively stimulating activities contribute to these differences. In females, the null associations could indicate a different pattern of brain aging or resilience.^70^ The lack of association between TV watching and brain structure in females may be related to different patterns of SB. Recent work found that when engaging in SB‐related TV watching, women in two cohorts tended to do so in a less prolonged and more intermittent manner than men.^71^, ^72^ This would result in shorter, interrupted periods of low‐cognitive‐demand SB compared to men, who may engage in more prolonged and uninterrupted passive activities.^71^, ^72^, ^73^ Furthermore, the lack of association between sitting during work in females and brain outcomes may be attributed to sex differences in the frequency of sitting. In our study, men were more likely to sit occasionally during work, which were consistently correlated with high cortical volume in various brain regions.

### 4.4 SB and brain regions

Sitting during work was positively associated with volume in the right frontal pole, a region implicated in prospective memory, planning, and abstract reasoning.^74^ This finding contrasts with the commonly reported adverse associations between less cognitively demanding activities, such as TV watching, and brain structure.^9^, ^65^ One possible explanation is that sitting during work hours often occurs in cognitively demanding contexts and involves sustained cognitive engagement, goal‐directed behavior, and decision‐making, which may help explain associations with the structural integrity of the frontal pole.^75^

Conversely, frequent TV watching was associated with lower volumes in regions involved in visual processing, emotion regulation, cognitive control, language, and the default mode network (including the precuneus), as well as the thalamus. These distributed associations suggest prolonged passive SB may relate to broad network‐level structural differences rather than a single system, potentially reflecting reduced cognitive engagement and cumulative vascular risk impacting multiple neural circuits. In addition, the thalamus has been linked to both vascular health and cognitive decline,^76^ and its reduced volume could reflect cumulative effects of vascular risk associated with a sedentary lifestyle.

### 4.5 Strengths and limitations

Strengths of our study include long follow‐up in a geographically diverse community‐based cohort of Black and White adults in the United States, which allowed us to explore the generalizability of previously reported associations between SB and brain structure. In addition, rigorously standardized sociodemographic and laboratory data provide a comprehensive evaluation of SB and brain structure. We conducted several sensitivity analyses, carefully assessing the role of potential mediators, pre‐existing cerebrovascular disease, *APOE* ɛ4 genotype, and current PA in our observed associations. The results of these sensitivity analyses demonstrated the robustness of our findings.

Our study also has some limitations. First, our findings were based on self‐reported instead of device‐measured SB, which may involve some recall bias. Although accelerometers provide a more accurate measure of total time in low acceleration activities, these devices provide limited granular insights into how SB in distinct contexts (e.g., TV watching and sitting during work) may influence brain structure. In addition, our findings are restricted to the frequency of time spent in context‐specific SB, which is aligned with previous recommendations on assessments using self‐reports that should focus on quantifying the time spent in specific forms of seated activities. Second, the questionnaire captures the perceived frequency of SBs within specific domains rather than their exact duration, preventing the establishment of precise time‐based cutoffs (e.g., hours/day) for each category and limiting direct quantification of sitting time. Third, the observational design limits causal inference, and self‐reported SB may be subject to recall bias. Finally, SB was not assessed at the imaging visit, preventing evaluation of changes over time; however, findings remained consistent when time‐varying SB from visits 1 and 3 was included in the models. Our findings provide insights into the potential long‐term implications of midlife sedentary patterns for later brain health.

Our findings indicate that TV watching and sitting at work are independent predictors of brain structure and WMH volume. Interventions targeting reductions in SB, particularly time in less cognitively demanding activities, may help preserve brain structure and reduce cerebrovascular risk. Moreover, public health strategies should consider the context and cognitive content of SB, as not all SBs may carry equal risk. Finally, tailored approaches that consider sex‐specific vulnerabilities may enhance the effectiveness of interventions aimed at promoting healthy brain aging.

## CONFLICT OF INTEREST STATEMENT

The authors declare no conflicts of interest. Author disclosures are available in the Supporting Information.

## CONSENT STATEMENT

The study was approved by institutional review boards at each study center, and all participants provided written informed consent to participate in each study visit and follow‐up.

## Supporting information




Supporting Information

Supporting Information
