# Supplementary material for: Associations of distinct sedentary behaviors with cortical, subcortical, and white matter hyperintensity volumes: Evidence from the ARIC study
Source: Alzheimers Dement. 2026 Jul 10;22(7):e71582. doi: 10.1002/alz.71582 (PMC13352356; doi:10.1002/alz.71582)
Supplement: Supplementary file 2 — Supporting Information [file ALZ-22-e71582-s001.docx]

**Supplemental Materials**

[**Supplementary Methods 1.** Classification of dementia, mild cognitive impairment, and cerebrovascular disease 3](#_Toc230440096)

[Neurocognitive Assessment and Diagnostic Review Process 3](#_Toc230440097)

[Classification of Dementia and MCI 3](#_Toc230440098)

[Cerebrovascular Disease-Related Cognitive Impairment 4](#_Toc230440099)

[**Supplementary Methods 2.** Regions of interest based on the Desikan-Killiany and subcortical segmentation atlases. 6](#_Toc230440100)

[**Supplementary Table 1.** Characteristics of the included participants by sex. ARIC study. N=1,712 7](#_Toc230440101)

[**Supplementary Table 2.** Association between context-specific sedentary behavior and cortical, subcortical, and white matter hyperintensity volumes. N=1,712. ARIC study. 8](#_Toc230440102)

[**Supplementary Table 3.** Sensitivity analysis on the association between sedentary behavior and cortical, subcortical, and white matter hyperintensity volumes additionally adjusting for physical activity at visit 5. N=1,712. ARIC study. 11](#_Toc230440103)

[**Supplementary Table 4.** Difference in regional brain volumes between the lowest (Never/Seldom) and the highest (Very often) frequency of TV watching. ARIC. N=1,712. 12](#_Toc230440104)

[**Supplementary Table 5.** Difference in regional brain volumes between the lowest (Never/Seldom) and the highest (Always) frequency of sitting during work. ARIC. N=1,712. 14](#_Toc230440105)

[**Supplementary Table 6.** Interaction between frequency of watching TV in leisure time and sex in the association with brain structure and white matter hyperintensity volume. N=1,712. ARIC study. 16](#_Toc230440106)

[**Supplementary Table 7.** Interaction between frequency of sitting during work and sex in the association with brain structure and white matter hyperintensity volume. N=1,712. ARIC study. 17](#_Toc230440107)

[**Supplementary Table 8.** Interaction between frequency of TV watching and sex in regional brain volumes between the lowest (Never/Seldom) and the highest (Very often) frequency of TV watching. ARIC. N=1,712. 18](#_Toc230440108)

[**Supplementary Table 9.** Interaction between frequency of sitting during work and sex in regional brain volumes between the lowest (Never/Seldom) and the highest (Always) frequency of sitting during work. ARIC. N=1,712. 20](#_Toc230440109)

[**Supplementary Table 10.** Sensitivity analysis on the association between sedentary behavior and cortical, subcortical, and white matter hyperintensity volumes, excluding participants with cerebrovascular disease-related MCI. N=1,612. ARIC study. 22](#_Toc230440110)

[**Supplementary Table 11.** Sensitivity analysis on the association between sedentary behavior, excluding participants with mild cognitive impairment. N=1,036. ARIC study. 23](#_Toc230440111)

[**Supplementary Table 12.** Sensitivity analysis on the association between sedentary behavior and cortical, subcortical, and white matter hyperintensity volumes, additionally adjusting for *APOE* ɛ4 genotype. N=822. ARIC study. 24](#_Toc230440112)

[**Supplementary Table 13.** Sensitivity analysis on the association between sedentary behavior and cortical, subcortical, and white matter hyperintensity volumes accounting for inverse probability attrition weighting. N=1,712. ARIC study. 25](#_Toc230440113)

[**Supplementary Table 14.** Sensitivity analysis on the association between time-fixed sedentary behavior and cortical, subcortical, and white matter hyperintensity volumes adjusting for time-varying covariates. N=1,712. ARIC study. 26](#_Toc230440114)

[**Supplementary Table 15.** Sensitivity analysis on the association between time-varying sedentary behavior and cortical, subcortical, and white matter hyperintensity volumes adjusting for time-varying covariates. N=1,712. ARIC study. 27](#_Toc230440115)

[**Supplementary Figure 1.** Association between brain structure and frequency of watching TV in leisure time (A) and sitting during work (B) in females. N=981. ARIC study. 28](#_Toc230440116)

[**Supplementary Figure 2.** Association between brain structure and the frequency of watching TV in leisure time (A) and sitting during work (B) in males. N=731. ARIC study. 29](#_Toc230440117)

[**Supplementary Figure 3.** Association between context-specific sedentary behavior and brain structure according to sex. N=1,712. ARIC study. 30](#_Toc230440118)

# **Supplementary Methods 1. Classification of dementia, mild cognitive impairment, and cerebrovascular disease**

The identification and classification of dementia, mild cognitive impairment (MCI), and cerebrovascular disease in the Atherosclerosis Risk in Communities Neurocognitive Study (ARIC-NCS) adhered to standardized procedures outlined in the ARIC-NCS manuals ([ARIC Manuals](https://aric.cscc.unc.edu/aric9/researchers/manuals)). Diagnoses were based on a comprehensive assessment that included cognitive testing, neurological examinations, clinical history, and neuroimaging data.

## Neurocognitive Assessment and Diagnostic Review Process

As previously described in detail, the ARIC-NCS protocol involved extensive neuropsychological testing across multiple domains, including memory, language, attention, and processing speed. Participants identified as being at risk for cognitive impairment, those who underwent MRI, and a random subsample of cognitively normal individuals completed an in-depth clinical evaluation. This included a neurological exam, the Clinical Dementia Rating (CDR) scale, the Functional Activities Questionnaire (FAQ), and the Neuropsychiatric Inventory. In addition to the primary ARIC-NCS neurocognitive visit, briefer cognitive assessments were conducted at visits 2 and 4. These earlier evaluations were used to detect cognitive decline and to inform the selection of participants for brain MRI at visit 5.

## Classification of Dementia and MCI

The classification of cognitive status - normal cognition, MCI, or dementia - was determined using a predefined algorithm. A complete description of the diagnostic algorithm used for classification is publicly available (<https://www5.cscc.unc.edu/aric9/sites/default/files/public/visitdocuments/v5/ARIC%20V5%20Derived%20Variable%20Dictionary.pdf>; page 81) and described in a previous publication.^1^ The algorithm incorporates cognitive performance across multiple domains, longitudinal cognitive change, and functional status measures (Diagnostic criteria included scores on the Mini-Mental State Examination (MMSE), CDR, and FAQ.

The algorithm defined cognitive impairment at visit 5 as either a low Mini-Mental State Examination score (≤21 for Whites and ≤19 for Blacks) or low performance (z score < –1.5 SD) in two or more cognitive domains (memory, executive function, language) combined with cognitive decline on at least one of the following tests: the Delayed Word Recall Test, the Digit Symbol Substitution Test, or the Word Fluency Test. Cognitive decline was defined as a visit 5 score minus the highest previous score falling below the 20th percentile on one test or below the 10th percentile on two or more tests. Dementia was defined by simultaneous evidence of cognitive impairment and significant functional impairment, indicated by a FAQ score greater than 5 or a CDR Sum of Boxes score exceeding 2.5.

Each participant's data was compiled into a comprehensive diagnostic packet. These packets were independently reviewed by both a physician (typically a neurologist or geriatrician) and a neuropsychologist. If the two reviewers disagreed on the diagnosis, a third clinical expert adjudicated the case. Final diagnoses were made in accordance with the DSM-IV^2^ and the criteria established by the National Institute on Aging–Alzheimer’s Association for MCI and dementia.^3,4^

## **Cerebrovascular Disease-Related Cognitive Impairment**

In one sensitivity analysis, we excluded 100 participants who were classified as having cerebrovascular disease-related MCI. This classification was based on modified NINDS-AIREN criteria^5^ and included three components:

1. **Clinical history**: Evidence of stroke temporally associated with cognitive decline, assessed using the Hachinski Ischemic Scale.
2. **Neuroimaging**: Presence of either bilateral or multiple infarcts, or extensive white matter hyperintensities (WMH). WMH burden was considered significant when >50% of the circumference of the corona radiata was affected or when ≥2 distinct infarcts were present.
3. **Neurological signs**: Physical examination findings indicating focal neurological deficits, such as asymmetric corticospinal tract dysfunction or classic post-stroke syndromes.

This rigorous, multi-tiered adjudication framework provided a consistent and validated approach for cognitive disorder classification in the ARIC-NCS.

**References**

1. Knopman DS, Gottesman RF, Sharrett AR, et al. Mild cognitive impairment and dementia prevalence: The Atherosclerosis Risk in Communities Neurocognitive Study. *Alzheimer’s and Dementia: Diagnosis, Assessment and Disease Monitoring*. 2016;2:1-11. doi:10.1016/J.DADM.2015.12.002,

2. Association AP. Diagnostic and Statistical Manual of Mental Disorders, Fourth Edition (DSM-IV) Washington. *DC, USA: American Psychiatric Association*. Published online 2004.

3. Albert MS, DeKosky ST, Dickson D, et al. The diagnosis of mild cognitive impairment due to Alzheimer’s disease: Recommendations from the National Institute on Aging-Alzheimer’s Association workgroups on diagnostic guidelines for Alzheimer’s disease. *Alzheimer’s & Dementia*. 2011;7(3):270-279. doi:10.1016/J.JALZ.2011.03.008

4. McKhann GM, Knopman DS, Chertkow H, et al. The diagnosis of dementia due to Alzheimer’s disease: Recommendations from the National Institute on Aging-Alzheimer’s Association workgroups on diagnostic guidelines for Alzheimer’s disease. *Alzheimer’s and Dementia*. 2011;7(3):263-269. doi:10.1016/J.JALZ.2011.03.005,

5. Van Straaten ECW, Scheltens P, Knol DL, et al. Operational Definitions for the NINDS-AIREN Criteria for Vascular Dementia. *Stroke*. 2003;34(8):1907-1912. doi:10.1161/01.STR.0000083050.44441.10

# **Supplementary Methods 2.** Regions of interest based on the Desikan-Killiany and subcortical segmentation atlases.

| **Brain region** | **Atlas** | **Grouped region** |
| --- | --- | --- |
| Amygdala | Subcortical segmentation | Temporal |
| Bankssts | Desikan-Killiany | Temporal |
| Caudal anterior cingulate | Desikan-Killiany | Frontal |
| Caudal middle frontal | Desikan-Killiany | Frontal |
| Caudate | Subcortical segmentation | Deep gray |
| Cuneus | Desikan-Killiany | Occipital |
| Entorhinal | Desikan-Killiany | Temporal |
| Frontal pole | Desikan-Killiany | Frontal |
| Fusiform | Desikan-Killiany | Temporal |
| Hippocampus | Subcortical segmentation | Temporal |
| Inferior parietal | Desikan-Killiany | Parietal |
| Inferior temporal | Desikan-Killiany | Temporal |
| Insula | Desikan-Killiany | Deep gray |
| Isthmus cingulate | Desikan-Killiany | Parietal |
| Lateral occipital | Desikan-Killiany | Occipital |
| Lateral orbitofrontal | Desikan-Killiany | Frontal |
| Lingual | Desikan-Killiany | Occipital |
| Medial orbitofrontal | Desikan-Killiany | Frontal |
| Middle temporal | Desikan-Killiany | Temporal |
| Pallidum | Subcortical segmentation | Deep gray |
| Paracentral | Desikan-Killiany | Frontal |
| Parahippocampal | Desikan-Killiany | Temporal |
| Pars opercularis | Desikan-Killiany | Frontal |
| Pars orbitalis | Desikan-Killiany | Frontal |
| Pars triangularis | Desikan-Killiany | Frontal |
| Pericalcarine | Desikan-Killiany | Occipital |
| Postcentral | Desikan-Killiany | Parietal |
| Posterior cingulate | Desikan-Killiany | Parietal |
| Precentral | Desikan-Killiany | Frontal |
| Precuneus | Desikan-Killiany | Parietal |
| Putamen | Subcortical segmentation | Deep gray |
| Rostral anterior cingulate | Desikan-Killiany | Frontal |
| Rostral middle frontal | Desikan-Killiany | Frontal |
| Superior frontal | Desikan-Killiany | Frontal |
| Superior parietal | Desikan-Killiany | Parietal |
| Superior temporal | Desikan-Killiany | Temporal |
| Supramarginal | Desikan-Killiany | Parietal |
| Temporal pole | Desikan-Killiany | Temporal |
| Thalamus proper | Subcortical segmentation | Deep gray |
| Transverse temporal | Desikan-Killiany | Temporal |

Note: ROIs were analyzed in both left and right hemispheres and plotted using the R package *ggseg (*version 1.6.5). Grouped regions were pre-defined and available in the ARIC neuroimaging dataset (<https://aric.cscc.unc.edu/aric9/sites/default/files/public/visitdocuments/v5/ARIC%20NCS%20Derived%20Variable%20Dictionary%20161031%20v1.pdf#page=19.09>). Bankssts, banks of the superior temporal sulcus.

# **Supplementary Table 1.** Characteristics of the included participants by sex. ARIC study. N=1,712

|  | Female (N=981) | Male (N=731) | P value |
| --- | --- | --- | --- |
| Age at visit 1 in years, mean (SD) | 53 (5.1) | 53 (5.3) | 0.120 |
| Age at visit 5 in years, mean (SD) | 76 (5.2) | 77 (5.3) | 0.074 |
| Race and ethnicity, n (%) |  |  | 0.040 |
| *Black* | 252 (25.7%) | 155 (21.2%) |  |
| *White* | 729 (74.3%) | 576 (78.8%) |  |
| Schooling (*< High School)*, n (%) | 163 (16.6%) | 88 (12.0%) | 0.010 |
| Race - Study center, n (%) |  |  | <0.001 |
| *Minnesota – White* | 215 (21.9%) | 221 (30.2%) |  |
| *Maryland – White* | 299 (30.5%) | 176 (24.1%) |  |
| *North Carolina – White* | 215 (21.9%) | 179 (24.5%) |  |
| *North Carolina – Black* | 12 (1.2%) | 10 (1.4%) |  |
| *Mississippi – Black* | 240 (24.5%) | 145 (19.8%) |  |
| Occupation, n (%) |  |  | <0.001 |
| *Managerial and Professional Specialty* | 203 (20.7%) | 282 (38.6%) |  |
| *Technical, Sales, and Administrative Support* | 295 (30.1%) | 134 (18.3%) |  |
| *Service Occupations* | 151 (15.4%) | 41 (5.6%) |  |
| *Farming, Forestry, and Fishing* | 0 (0%) | 5 (0.7%) |  |
| *Precision Production, Craft, and Repair* | 31 (3.2%) | 92 (12.6%) |  |
| *Operators, Fabricators, and Laborers* | 56 (5.7%) | 83 (11.4%) |  |
| *Homemakers* | 150 (15.3%) | 0 (0%) |  |
| *Retired* | 67 (6.8%) | 94 (12.9%) |  |
| *Other* | 28 (2.9%) | 0 (0%) |  |
| Current smoking, n (%) |  |  | <0.001 |
| *Current smoker* | 201 (20.5%) | 132 (18.1%) |  |
| *Former smoker* | 212 (21.6%) | 340 (46.5%) |  |
| *Never smoker* | 568 (57.9%) | 259 (35.4%) |  |
| Alcohol consumption, n (%) |  |  | <0.001 |
| *Current Drinker* | 543 (55.4%) | 505 (69.1%) |  |
| *Former Drinker* | 130 (13.3%) | 104 (14.2%) |  |
| *Never Drinker* | 308 (31.4%) | 113 (15.5%) |  |
| Physical activity score | 2.4 (0.76) | 2.7 (0.87) | <0.001 |
| Body mass index, n (%) | 27 (5.4) | 27 (3.3) | <0.001 |
| *Underweight* | 8 (0.8%) | 1 (0.1%) |  |
| *Normal weight* | 387 (39.4%) | 199 (27.2%) |  |
| *Overweight* | 289 (29.5%) | 409 (56.0%) |  |
| *Obese* | 297 (30.3%) | 122 (16.7%) |  |
| Hypertension (yes), n (%) | 262 (26.7%) | 160 (21.9%) | 0.026 |
| Diabetes (yes), n (%) | 53 (5.4%) | 47 (6.4%) | 0.448 |
| TV watching, n (%) |  |  | 0.022 |
| *Never/Seldom* | 201 (20.5%) | 109 (14.9%) |  |
| *Sometimes* | 455 (46.4%) | 369 (50.5%) |  |
| *Often* | 268 (27.3%) | 215 (29.4%) |  |
| *Very often* | 57 (5.8%) | 38 (5.2%) |  |
| Sitting during work, n (%) |  |  | <0.001 |
| *Never/Seldom* | 265 (27.0%) | 152 (20.8%) |  |
| *Sometimes* | 209 (21.3%) | 203 (27.8%) |  |
| *Often* | 185 (18.9%) | 253 (34.6%) |  |
| *Always* | 136 (13.9%) | 67 (9.2%) |  |
| *Unemployed* | 186 (19.0%) | 56 (7.7%) |  |

All variables were assessed at visit 1. Age was also assessed at visit 5. SD: standard deviation

# **Supplementary Table 2.** Association between context-specific sedentary behavior and cortical, subcortical, and white matter hyperintensity volumes. N=1,712. ARIC study.

|  | TV watching | | | |  | Sitting during work | | | | |
| --- | --- | --- | --- | --- | --- | --- | --- | --- | --- | --- |
| Brain regions | *Never/*  *seldom* | *Sometimes* | *Often* | *Very often* | *Never/*  *seldom* | *Sometimes* | *Often* | *Always* | *Do not work* |  |
| AD signature |  |  |  |  |  |  |  |  |  |  |
| Crude model | 1.00 | -0.03 (-0.12, 0.06) | -0.1 (-0.2, -0.01) | **-0.42 (-0.59, -0.26)** | 1.00 | 0.08 (-0.02, 0.17) | **0.19 (0.1, 0.29)** | **0.2 (0.08, 0.32)** | -0.12 (-0.24, 0) |  |
| Minimally adjusted | 1.00 | 0.01 (-0.07, 0.09) | -0.04 (-0.13, 0.04) | **-0.24 (-0.39, -0.09)** | 1.00 | 0.04 (-0.05, 0.13) | 0.08 (-0.01, 0.17) | 0.11 (0, 0.22) | -0.03 (-0.17, 0.1) |  |
| Adjusted | 1.00 | 0.02 (-0.06, 0.09) | -0.03 (-0.12, 0.06) | **-0.25 (-0.4, -0.1)** | 1.00 | 0.06 (-0.03, 0.15) | 0.08 (-0.01, 0.17) | 0.12 (0.01, 0.23) | 0.01 (-0.12, 0.14) |  |
| Fully adjusted | 1.00 | 0.02 (-0.06, 0.1) | -0.03 (-0.12, 0.06) | **-0.24 (-0.39, -0.09)** | 1.00 | 0.06 (-0.03, 0.15) | 0.08 (-0.01, 0.17) | 0.12 (0.01, 0.23) | 0 (-0.13, 0.13) |  |
| *Frontal CV* |  |  |  |  |  |  |  |  |  |  |
| Crude model | 1.00 | -0.08 (-0.16, 0) | **-0.16 (-0.25, -0.08)** | **-0.38 (-0.53, -0.24)** | 1.00 | **0.13 (0.04, 0.21)** | **0.24 (0.16, 0.32)** | **0.19 (0.09, 0.29)** | -0.04 (-0.14, 0.07) |  |
| Minimally adjusted | 1.00 | -0.05 (-0.13, 0.02) | **-0.11 (-0.2, -0.03)** | **-0.25 (-0.39, -0.11)** | 1.00 | **0.11 (0.03, 0.19)** | **0.18 (0.09, 0.26)** | **0.13 (0.03, 0.23)** | -0.01 (-0.13, 0.12) |  |
| Adjusted | 1.00 | -0.05 (-0.13, 0.02) | -0.1 (-0.19, -0.02) | **-0.26 (-0.4, -0.12)** | 1.00 | **0.12 (0.03, 0.2)** | **0.18 (0.1, 0.27)** | **0.14 (0.04, 0.24)** | 0.03 (-0.09, 0.15) |  |
| Fully adjusted | 1.00 | -0.05 (-0.13, 0.02) | -0.1 (-0.19, -0.02) | **-0.25 (-0.4, -0.11)** | 1.00 | **0.12 (0.03, 0.2)** | **0.18 (0.1, 0.26)** | **0.14 (0.04, 0.24)** | 0.03 (-0.09, 0.15) |  |
| *Temporal CV* |  |  |  |  |  |  |  |  |  |  |
| Crude model | 1.00 | -0.05 (-0.14, 0.03) | -0.11 (-0.2, -0.01) | **-0.29 (-0.45, -0.13)** | 1.00 | **0.18 (0.09, 0.28)** | **0.23 (0.14, 0.33)** | **0.17 (0.05, 0.28)** | -0.1 (-0.22, 0.01) |  |
| Minimally adjusted | 1.00 | -0.03 (-0.11, 0.05) | -0.07 (-0.16, 0.02) | -0.17 (-0.32, -0.02) | 1.00 | **0.15 (0.06, 0.24)** | **0.15 (0.06, 0.24)** | 0.1 (0, 0.21) | 0.01 (-0.11, 0.14) |  |
| Adjusted | 1.00 | -0.03 (-0.1, 0.05) | -0.04 (-0.13, 0.05) | -0.15 (-0.3, 0) | 1.00 | **0.16 (0.07, 0.24)** | **0.15 (0.06, 0.24)** | 0.12 (0.01, 0.22) | 0.03 (-0.1, 0.16) |  |
| Fully adjusted | 1.00 | -0.02 (-0.1, 0.06) | -0.04 (-0.12, 0.05) | -0.14 (-0.29, 0.01) | 1.00 | **0.16 (0.07, 0.24)** | **0.15 (0.06, 0.23)** | 0.12 (0.01, 0.23) | 0.02 (-0.1, 0.15) |  |
| *Occipital CV* |  |  |  |  |  |  |  |  |  |  |
| Crude model | 1.00 | **-0.15 (-0.25, -0.06)** | -0.11 (-0.22, -0.01) | **-0.44 (-0.62, -0.26)** | 1.00 | **0.16 (0.06, 0.27)** | **0.24 (0.14, 0.35)** | **0.3 (0.18, 0.43)** | 0.06 (-0.07, 0.19) |  |
| Minimally adjusted | 1.00 | -0.1 (-0.19, -0.01) | -0.02 (-0.12, 0.08) | -0.19 (-0.36, -0.02) | 1.00 | 0.11 (0.01, 0.21) | 0.09 (-0.01, 0.19) | **0.22 (0.1, 0.34)** | 0.11 (-0.04, 0.25) |  |
| Adjusted | 1.00 | -0.1 (-0.18, -0.01) | -0.01 (-0.11, 0.09) | -0.2 (-0.37, -0.03) | 1.00 | **0.13 (0.03, 0.23)** | 0.11 (0.01, 0.21) | **0.22 (0.1, 0.34)** | 0.14 (-0.01, 0.28) |  |
| Fully adjusted | 1.00 | -0.1 (-0.19, -0.01) | -0.02 (-0.12, 0.08) | **-0.21 (-0.38, -0.04)** | 1.00 | **0.13 (0.03, 0.23)** | 0.11 (0.01, 0.21) | **0.22 (0.09, 0.34)** | 0.14 (0, 0.29) |  |
| *Parietal CV* |  |  |  |  |  |  |  |  |  |  |
| Crude model | 1.00 | -0.03 (-0.11, 0.05) | -0.13 (-0.23, -0.04) | **-0.39 (-0.54, -0.24)** | 1.00 | **0.14 (0.05, 0.23)** | **0.24 (0.15, 0.32)** | **0.27 (0.16, 0.37)** | 0 (-0.11, 0.11) |  |
| Minimally adjusted | 1.00 | 0.02 (-0.05, 0.1) | -0.05 (-0.14, 0.03) | -0.15 (-0.3, -0.01) | 1.00 | 0.11 (0.02, 0.19) | **0.12 (0.03, 0.2)** | **0.17 (0.07, 0.28)** | 0.04 (-0.08, 0.16) |  |
| Adjusted | 1.00 | 0.03 (-0.05, 0.1) | -0.04 (-0.13, 0.04) | -0.17 (-0.31, -0.03) | 1.00 | **0.12 (0.03, 0.2)** | **0.12 (0.03, 0.2)** | **0.18 (0.08, 0.29)** | 0.08 (-0.04, 0.2) |  |
| Fully adjusted | 1.00 | 0.03 (-0.04, 0.11) | -0.04 (-0.12, 0.04) | -0.16 (-0.3, -0.02) | 1.00 | **0.12 (0.03, 0.2)** | **0.11 (0.03, 0.2)** | **0.19 (0.08, 0.29)** | 0.08 (-0.04, 0.2) |  |
| *Subcortical deep grey volume* |  |  |  |  |  |  |  |  |  |  |
| Crude model | 1.00 | -0.02 (-0.12, 0.07) | -0.05 (-0.16, 0.06) | -0.13 (-0.32, 0.05) | 1.00 | -0.02 (-0.13, 0.08) | -0.01 (-0.11, 0.09) | 0.07 (-0.05, 0.2) | -0.17 (-0.3, -0.04) |  |
| Minimally adjusted | 1.00 | -0.02 (-0.11, 0.08) | -0.04 (-0.15, 0.07) | -0.12 (-0.3, 0.07) | 1.00 | -0.02 (-0.13, 0.08) | -0.01 (-0.11, 0.1) | 0.06 (-0.07, 0.19) | -0.05 (-0.2, 0.11) |  |
| Adjusted | 1.00 | 0.01 (-0.09, 0.11) | -0.01 (-0.12, 0.1) | -0.1 (-0.28, 0.09) | 1.00 | 0 (-0.11, 0.11) | -0.01 (-0.11, 0.1) | 0.09 (-0.04, 0.22) | -0.03 (-0.19, 0.13) |  |
| Fully adjusted | 1.00 | 0 (-0.09, 0.1) | -0.02 (-0.13, 0.09) | -0.11 (-0.3, 0.07) | 1.00 | 0 (-0.11, 0.11) | 0 (-0.11, 0.11) | 0.08 (-0.05, 0.21) | -0.02 (-0.18, 0.14) |  |
| *WMH volume* |  |  |  |  |  |  |  |  |  |  |
| Crude model | 1.00 | 0.13 (0.01, 0.25) | **0.18 (0.04, 0.31)** | **0.67 (0.44, 0.89)** | 1.00 | **-0.18 (-0.32, -0.05)** | -0.13 (-0.26, 0) | **-0.26 (-0.42, -0.1)** | 0.24 (0.08, 0.4) |  |
| Minimally adjusted | 1.00 | 0.12 (0.01, 0.23) | 0.11 (-0.02, 0.23) | **0.52 (0.31, 0.74)** | 1.00 | -0.13 (-0.26, 0) | 0.01 (-0.12, 0.14) | **-0.23 (-0.38, -0.07)** | 0.06 (-0.13, 0.24) |  |
| Adjusted | 1.00 | 0.1 (-0.01, 0.21) | 0.09 (-0.04, 0.21) | **0.52 (0.31, 0.74)** | 1.00 | -0.14 (-0.27, -0.01) | 0 (-0.13, 0.13) | **-0.26 (-0.41, -0.1)** | 0.01 (-0.18, 0.19) |  |
| Fully adjusted | 1.00 | 0.1 (-0.01, 0.21) | 0.08 (-0.04, 0.21) | **0.52 (0.3, 0.74)** | 1.00 | -0.14 (-0.27, -0.01) | 0 (-0.13, 0.13) | **-0.26 (-0.41, -0.1)** | 0.01 (-0.18, 0.2) |  |

Values are beta coefficients and respective 95% confidence intervals extracted from multivariate generalized linear models.

**Bold values indicate false discovery rate-adjusted p<0.05**

Crude model: adjusted only for estimated total intracranial volume at visit 5.

Minimally adjusted: additionally adjusted for gender, age (visit 5), race-center, education, and occupation.

Adjusted: additionally adjusted for smoking, drinking, BMI, diabetes, and hypertension at visit 1, and mutually adjusted for frequency of TV and sitting during work.

Fully adjusted: additionally adjusted for physical activity at visit 1.

# **Supplementary Table 3.** Sensitivity analysis on the association between sedentary behavior and cortical, subcortical, and white matter hyperintensity volumes additionally adjusting for physical activity at visit 5. N=1,712. ARIC study.

|  | TV watching | | | | Sitting during work | | | | |
| --- | --- | --- | --- | --- | --- | --- | --- | --- | --- |
| Brain region | Never/  seldom | Sometimes | Often | Very often | Never/  seldom | Sometimes | Often | Always | Do not work |
| *AD signature* | 1.00 | 0.02 (-0.06, 0.1) | -0.01 (-0.1, 0.07) | **-0.21 (-0.36, -0.06)** | 1.00 | 0.05 (-0.04, 0.14) | 0.09 (0, 0.18) | 0.11 (0, 0.21) | 0.02 (-0.11, 0.15) |
| *Frontal CV* | 1.00 | -0.06 (-0.13, 0.02) | -0.09 (-0.17, 0) | **-0.24 (-0.39, -0.1)** | 1.00 | **0.1 (0.02, 0.19)** | **0.18 (0.09, 0.26)** | **0.13 (0.03, 0.23)** | 0.04 (-0.09, 0.16) |
| *Temporal CV* | 1.00 | -0.03 (-0.11, 0.04) | -0.03 (-0.12, 0.06) | -0.13 (-0.28, 0.02) | 1.00 | **0.14 (0.06, 0.23)** | **0.15 (0.06, 0.24)** | 0.11 (0, 0.22) | 0.05 (-0.08, 0.17) |
| *Occipital CV* | 1.00 | -0.11 (-0.2, -0.02) | -0.01 (-0.11, 0.09) | **-0.21 (-0.39, -0.04)** | 1.00 | **0.12 (0.02, 0.22)** | 0.1 (0, 0.2) | **0.2 (0.08, 0.32)** | 0.13 (-0.01, 0.28) |
| *Parietal CV* | 1.00 | 0.02 (-0.05, 0.1) | -0.03 (-0.11, 0.05) | -0.13 (-0.28, 0.01) | 1.00 | **0.11 (0.02, 0.19)** | **0.12 (0.04, 0.21)** | **0.17 (0.07, 0.27)** | 0.1 (-0.03, 0.22) |
| *Deep gray CV* | 1.00 | -0.02 (-0.11, 0.08) | -0.04 (-0.14, 0.07) | -0.13 (-0.32, 0.06) | 1.00 | 0.01 (-0.1, 0.11) | 0.02 (-0.09, 0.13) | 0.09 (-0.04, 0.23) | 0 (-0.16, 0.16) |
| *WMH volume* | 1.00 | 0.1 (-0.02, 0.21) | 0.05 (-0.08, 0.18) | **0.48 (0.26, 0.69)** | 1.00 | -0.13 (-0.25, 0) | 0 (-0.13, 0.12) | **-0.24 (-0.39,**  **-0.08)** | 0.01 (-0.17, 0.2) |

Values are beta coefficients and respective 95% confidence intervals extracted from multivariate generalized linear models.

**Bold values indicate false discovery rate-adjusted p<0.05**

Generalized linear models adjusted for sex, age (visit 5), race-center, education, occupation, BMI, diabetes, hypertension, estimated total intracranial volume at visit 5, physical activity at visit 1 and 5, and mutually adjusted for frequency of TV and sitting during work.

# **Supplementary Table 4.** Difference in regional brain volumes between the lowest (Never/Seldom) and the highest (Very often) frequency of TV watching. ARIC. N=1,712.

| **Brain region** | **Hemisphere** | **Beta (mm^3^)** | **P-value** | **FDR P-value** |
| --- | --- | --- | --- | --- |
| Amygdala | Left | 29.10 | 0.209 | 0.378 |
| Bankssts | Left | -1.05 | 0.981 | 0.993 |
| Caudal anterior cingulate | Left | -49.60 | 0.293 | 0.426 |
| Caudal middle frontal | Left | -83.98 | 0.382 | 0.509 |
| Caudate | Left | -16.14 | 0.788 | 0.840 |
| Cuneus | Left | -50.04 | 0.268 | 0.407 |
| Entorhinal | Left | -42.66 | 0.270 | 0.407 |
| Frontal pole | Left | 4.79 | 0.769 | 0.831 |
| Fusiform | Left | -49.93 | 0.704 | 0.793 |
| Hippocampus | Left | -43.41 | 0.404 | 0.513 |
| Inferior parietal | Left | -181.30 | 0.256 | 0.407 |
| Inferior temporal | Left | -77.42 | 0.601 | 0.697 |
| Insula | Left | -181.94 | 0.012 | 0.054 |
| Isthmus cingulate | Left | -124.62 | 0.003 | 0.027 |
| Lateral occipital | Left | -143.01 | 0.328 | 0.460 |
| Lateral orbitofrontal | Left | -181.21 | 0.010 | 0.052 |
| Lingual | Left | -37.81 | 0.736 | 0.817 |
| Medial orbitofrontal | Left | -76.18 | 0.193 | 0.358 |
| Middle temporal | Left | -0.80 | 0.995 | 0.995 |
| Pallidum | Left | -32.68 | 0.164 | 0.312 |
| Paracentral | Left | -62.78 | 0.228 | 0.380 |
| Parahippocampal | Left | -60.52 | 0.108 | 0.216 |
| Pars opercularis | Left | -143.70 | 0.059 | 0.151 |
| Pars orbitalis | Left | -99.51 | 0.001 | 0.015 |
| Pars triangularis | Left | -240.64 | 0.000 | 0.002 |
| Pericalcarine | Left | -83.88 | 0.031 | 0.098 |
| Postcentral | Left | -301.05 | 0.014 | 0.058 |
| Posterior cingulate | Left | -134.73 | 0.006 | 0.044 |
| Precentral | Left | -225.42 | 0.093 | 0.193 |
| Precuneus | Left | -257.03 | 0.008 | 0.047 |
| Putamen | Left | -1.90 | 0.978 | 0.993 |
| Rostral anterior cingulate | Left | -160.83 | 0.001 | 0.015 |
| Rostral middle frontal | Left | -479.24 | 0.002 | 0.023 |
| Superior frontal | Left | -179.53 | 0.342 | 0.464 |
| Superior parietal | Left | -103.32 | 0.503 | 0.609 |
| Superior temporal | Left | -273.71 | 0.024 | 0.086 |
| Supramarginal | Left | -103.08 | 0.438 | 0.547 |
| Temporal pole | Left | -94.48 | 0.035 | 0.105 |
| Thalamus proper | Left | -108.73 | 0.059 | 0.151 |
| Transverse temporal | Left | -60.91 | 0.007 | 0.045 |
| Amygdala | Right | -12.79 | 0.554 | 0.651 |
| Bankssts | Right | -38.74 | 0.340 | 0.464 |
| Caudal anterior cingulate | Right | 93.81 | 0.068 | 0.160 |
| Caudal middle frontal | Right | -332.15 | 0.001 | 0.015 |
| Caudate | Right | 49.13 | 0.388 | 0.509 |
| Cuneus | Right | -111.08 | 0.032 | 0.098 |
| Entorhinal | Right | -49.60 | 0.238 | 0.388 |
| Frontal pole | Right | -24.29 | 0.222 | 0.378 |
| Fusiform | Right | 77.75 | 0.543 | 0.648 |
| Hippocampus | Right | -43.37 | 0.402 | 0.513 |
| Inferior parietal | Right | -330.90 | 0.070 | 0.160 |
| Inferior temporal | Right | -381.10 | 0.012 | 0.054 |
| Insula | Right | -80.63 | 0.291 | 0.426 |
| Isthmus cingulate | Right | -108.64 | 0.005 | 0.042 |
| Lateral occipital | Right | -275.39 | 0.064 | 0.155 |
| Lateral orbitofrontal | Right | -97.42 | 0.159 | 0.311 |
| Lingual | Right | -221.27 | 0.028 | 0.096 |
| Medial orbitofrontal | Right | -110.45 | 0.054 | 0.150 |
| Middle temporal | Right | -280.71 | 0.031 | 0.098 |
| Pallidum | Right | -61.91 | 0.002 | 0.023 |
| Paracentral | Right | -72.20 | 0.218 | 0.378 |
| Parahippocampal | Right | -57.07 | 0.094 | 0.193 |
| Pars opercularis | Right | -158.05 | 0.016 | 0.065 |
| Pars orbitalis | Right | -98.94 | 0.010 | 0.052 |
| Pars triangularis | Right | -124.17 | 0.072 | 0.160 |
| Pericalcarine | Right | -165.37 | 0.000 | 0.005 |
| Postcentral | Right | 205.86 | 0.077 | 0.167 |
| Posterior cingulate | Right | -31.13 | 0.494 | 0.608 |
| Precentral | Right | 13.24 | 0.925 | 0.974 |
| Precuneus | Right | -236.24 | 0.019 | 0.074 |
| Putamen | Right | -31.92 | 0.650 | 0.743 |
| Rostral anterior cingulate | Right | 47.10 | 0.269 | 0.407 |
| Rostral middle frontal | Right | -330.36 | 0.041 | 0.118 |
| Superior frontal | Right | -511.82 | 0.007 | 0.045 |
| Superior parietal | Right | 10.47 | 0.946 | 0.983 |
| Superior temporal | Right | -140.76 | 0.221 | 0.378 |
| Supramarginal | Right | -39.93 | 0.762 | 0.831 |
| Temporal pole | Right | -40.03 | 0.322 | 0.460 |
| Thalamus proper | Right | -163.38 | 0.003 | 0.030 |
| Transverse temporal | Right | -32.42 | 0.061 | 0.153 |

Generalized linear models adjusted for gender, age (visit 5), race-center, education, occupation, BMI, diabetes, hypertension, visit 1, physical activity, estimated total intracranial volume at visit 5, and frequency of sitting during work. Bankssts, banks of the superior temporal sulcus.

# **Supplementary Table 5.** Difference in regional brain volumes between the lowest (Never/Seldom) and the highest (Always) frequency of sitting during work. ARIC. N=1,712.

| **Brain region** | **Hemisphere** | **Beta (mm^3^)** | **P-value** | **FDR P-value** |
| --- | --- | --- | --- | --- |
| Amygdala | Left | 8.21 | 0.615 | 0.747 |
| Bankssts | Left | 4.72 | 0.880 | 0.880 |
| Caudal anterior cingulate | Left | 13.79 | 0.679 | 0.772 |
| Caudal middle frontal | Left | 21.01 | 0.756 | 0.818 |
| Caudate | Left | 44.86 | 0.289 | 0.432 |
| Cuneus | Left | 60.94 | 0.056 | 0.145 |
| Entorhinal | Left | 12.01 | 0.660 | 0.765 |
| Frontal pole | Left | 15.48 | 0.178 | 0.323 |
| Fusiform | Left | -46.47 | 0.616 | 0.747 |
| Hippocampus | Left | 37.69 | 0.304 | 0.434 |
| Inferior parietal | Left | 253.06 | 0.025 | 0.094 |
| Inferior temporal | Left | 157.31 | 0.132 | 0.257 |
| Insula | Left | -105.95 | 0.038 | 0.114 |
| Isthmus cingulate | Left | 77.91 | 0.008 | 0.052 |
| Lateral occipital | Left | 347.57 | 0.001 | 0.020 |
| Lateral orbitofrontal | Left | 154.11 | 0.002 | 0.021 |
| Lingual | Left | 174.82 | 0.027 | 0.098 |
| Medial orbitofrontal | Left | 71.42 | 0.083 | 0.202 |
| Middle temporal | Left | 148.59 | 0.118 | 0.245 |
| Pallidum | Left | -4.35 | 0.793 | 0.841 |
| Paracentral | Left | 71.46 | 0.052 | 0.138 |
| Parahippocampal | Left | -54.51 | 0.040 | 0.114 |
| Pars opercularis | Left | 61.29 | 0.253 | 0.404 |
| Pars orbitalis | Left | 44.65 | 0.035 | 0.114 |
| Pars triangularis | Left | 35.37 | 0.380 | 0.507 |
| Pericalcarine | Left | 57.26 | 0.037 | 0.114 |
| Postcentral | Left | 149.03 | 0.083 | 0.202 |
| Posterior cingulate | Left | 53.84 | 0.119 | 0.245 |
| Precentral | Left | 304.15 | 0.001 | 0.021 |
| Precuneus | Left | 72.35 | 0.292 | 0.432 |
| Putamen | Left | -63.54 | 0.200 | 0.348 |
| Rostral anterior cingulate | Left | -31.96 | 0.347 | 0.479 |
| Rostral middle frontal | Left | 52.29 | 0.632 | 0.754 |
| Superior frontal | Left | 335.50 | 0.012 | 0.059 |
| Superior parietal | Left | 296.65 | 0.006 | 0.050 |
| Superior temporal | Left | 267.77 | 0.002 | 0.021 |
| Supramarginal | Left | 219.74 | 0.019 | 0.076 |
| Temporal pole | Left | 65.49 | 0.039 | 0.114 |
| Thalamus proper | Left | 103.49 | 0.011 | 0.059 |
| Transverse temporal | Left | 12.06 | 0.448 | 0.588 |
| Amygdala | Right | -36.65 | 0.016 | 0.071 |
| Bankssts | Right | 37.25 | 0.194 | 0.344 |
| Caudal anterior cingulate | Right | 71.10 | 0.050 | 0.137 |
| Caudal middle frontal | Right | 84.83 | 0.212 | 0.361 |
| Caudate | Right | 56.02 | 0.163 | 0.305 |
| Cuneus | Right | 27.15 | 0.456 | 0.589 |
| Entorhinal | Right | -6.52 | 0.826 | 0.858 |
| Frontal pole | Right | 60.48 | 0.000 | 0.001 |
| Fusiform | Right | 79.68 | 0.376 | 0.507 |
| Hippocampus | Right | -9.29 | 0.799 | 0.841 |
| Inferior parietal | Right | 212.20 | 0.100 | 0.235 |
| Inferior temporal | Right | 161.18 | 0.132 | 0.257 |
| Insula | Right | -131.71 | 0.014 | 0.068 |
| Isthmus cingulate | Right | 9.63 | 0.725 | 0.795 |
| Lateral occipital | Right | 323.13 | 0.002 | 0.021 |
| Lateral orbitofrontal | Right | 168.81 | 0.001 | 0.020 |
| Lingual | Right | 182.54 | 0.010 | 0.059 |
| Medial orbitofrontal | Right | 64.62 | 0.110 | 0.245 |
| Middle temporal | Right | 271.13 | 0.003 | 0.029 |
| Pallidum | Right | -5.56 | 0.692 | 0.772 |
| Paracentral | Right | -45.15 | 0.275 | 0.423 |
| Parahippocampal | Right | 13.33 | 0.579 | 0.724 |
| Pars opercularis | Right | 33.44 | 0.470 | 0.597 |
| Pars orbitalis | Right | -4.21 | 0.877 | 0.880 |
| Pars triangularis | Right | 48.92 | 0.314 | 0.441 |
| Pericalcarine | Right | 48.81 | 0.109 | 0.245 |
| Postcentral | Right | 114.41 | 0.164 | 0.305 |
| Posterior cingulate | Right | 12.59 | 0.695 | 0.772 |
| Precentral | Right | 212.04 | 0.033 | 0.114 |
| Precuneus | Right | 170.26 | 0.017 | 0.071 |
| Putamen | Right | -8.21 | 0.868 | 0.880 |
| Rostral anterior cingulate | Right | 13.72 | 0.648 | 0.762 |
| Rostral middle frontal | Right | 117.78 | 0.302 | 0.434 |
| Superior frontal | Right | 164.64 | 0.221 | 0.369 |
| Superior parietal | Right | 293.30 | 0.007 | 0.050 |
| Superior temporal | Right | 127.37 | 0.116 | 0.245 |
| Supramarginal | Right | 294.79 | 0.002 | 0.021 |
| Temporal pole | Right | 33.83 | 0.236 | 0.385 |
| Thalamus proper | Right | 99.57 | 0.011 | 0.059 |
| Transverse temporal | Right | 13.33 | 0.275 | 0.423 |

Generalized linear models adjusted for gender, age (visit 5), race-center, education, occupation, BMI, diabetes, hypertension, visit 1, physical activity, estimated total intracranial volume at visit 5, and frequency of TV. Bankssts, banks of the superior temporal sulcus.

# **Supplementary Table 6.** Interaction between frequency of watching TV in leisure time and sex in the association with brain structure and white matter hyperintensity volume. N=1,712. ARIC study.

|  | Beta (mm3) | FDR P-value |
| --- | --- | --- |
| AD signature regions |  |  |
| Main effects |  |  |
| Frequency of TV watching | 176.404 (-140.794, 493.603) | 0.368 |
| Sex | 2823.887 (1555.71, 4092.063) | <0.001 |
| Interaction term | -1249.393 (-1753.842, -744.944) | <0.001 |
| Frontal regions |  |  |
| Main effects |  |  |
| Frequency of TV watching | 40.438 (-650.242, 731.117) | 0.938 |
| Sex | 4308.745 (1547.374, 7070.116) | 0.006 |
| Interaction term | -2718.7 (-3817.104, -1620.295) | <0.001 |
| Temporal region |  |  |
| Main effects |  |  |
| Frequency of TV watching | 353.831 (-166.964, 874.626) | 0.257 |
| Sex | 5024.267 (2942.102, 7106.432) | <0.001 |
| Interaction term | -1772.697 (-2600.93, -944.463) | <0.001 |
| Occipital region |  |  |
| Main effects |  |  |
| Frequency of TV watching | 154.696 (-137.869, 447.262) | 0.392 |
| Sex | 2255.142 (1085.451, 3424.833) | 0.001 |
| Interaction term | -731.47 (-1196.744, -266.196) | 0.005 |
| Parietal region |  |  |
| Main effects |  |  |
| Frequency of TV watching | 242.472 (-305.95, 790.893) | 0.475 |
| Sex | 2698.225 (505.608, 4890.841) | 0.028 |
| Interaction term | -1907.626 (-2779.794, -1035.458) | <0.001 |
| Deep gray subcortical region |  |  |
| Main effects |  |  |
| Frequency of TV watching | 165.645 (-13.221, 344.511) | 0.139 |
| Sex | 1153.124 (438.009, 1868.239) | 0.009 |
| Interaction term | -614.955 (-899.41, -330.501) | <0.001 |
| White matter hyperintensity |  |  |
| Main effects |  |  |
| Frequency of TV watching | 0.059 (0.001, 0.118) | 0.099 |
| Sex | -0.39 (-0.624, -0.156) | 0.004 |
| Interaction term | 0.042 (-0.051, 0.135) | 0.495 |

Generalized linear models included race-center, occupational status, schooling, body mass index, diabetes, hypertension, physical activity at visit 1, age and intracranial volume at visit 5, and the frequency of sitting during work. Alzheimer’s disease (AD) signature regions include volume of the parahippocampal, entorhinal, inferior parietal lobules, hippocampus, and precuneus.

# **Supplementary Table 7.** Interaction between frequency of sitting during work and sex in the association with brain structure and white matter hyperintensity volume. N=1,712. ARIC study.

|  | Beta (mm3) | FDR P-value |
| --- | --- | --- |
| AD signature regions |  |  |
| Main effects |  |  |
| Frequency of sitting during work | 132.987 (-68.442, 334.416) | 0.320 |
| Sex | 264.664 (-791.662, 1320.99) | 0.772 |
| Interaction term | -67.377 (-413.11, 278.357) | 0.786 |
| Frontal regions |  |  |
| Main effects |  |  |
| Frequency of sitting during work | 109.783 (-330.393, 549.959) | 0.758 |
| Sex | -3650.295 (-5958.648, -1341.941) | 0.006 |
| Interaction term | 893.275 (137.755, 1648.795) | 0.049 |
| Temporal region |  |  |
| Main effects |  |  |
| Frequency of sitting during work | 203.012 (-128.842, 534.866) | 0.397 |
| Sex | 1557.781 (-182.514, 3298.076) | 0.188 |
| Interaction term | -92.74 (-662.336, 476.855) | 0.801 |
| Occipital region |  |  |
| Main effects |  |  |
| Frequency of sitting during work | 176.339 (-8.561, 361.239) | 0.106 |
| Sex | 183.847 (-785.799, 1153.494) | 0.745 |
| Interaction term | 204.027 (-113.337, 521.39) | 0.322 |
| Parietal region |  |  |
| Main effects |  |  |
| Frequency of sitting during work | 253.569 (-94.514, 601.653) | 0.251 |
| Sex | -2674.097 (-4499.505, -848.688) | 0.010 |
| Interaction term | 476.123 (-121.33, 1073.576) | 0.204 |
| Deep gray subcortical region |  |  |
| Main effects |  |  |
| Frequency of sitting during work | 22.707 (-90.849, 136.264) | 0.824 |
| Sex | -247.102 (-842.61, 348.405) | 0.586 |
| Interaction term | 8.938 (-185.97, 203.847) | 0.959 |
| White matter hyperintensity |  |  |
| Main effects |  |  |
| Frequency of sitting during work | -0.015 (-0.052, 0.022) | 0.532 |
| Sex | -0.282 (-0.476, -0.087) | 0.011 |
| Interaction term | -0.007 (-0.07, 0.057) | 0.868 |

Generalized linear models included race-center, occupational status, schooling, body mass index, diabetes, hypertension, physical activity at visit 1, age and intracranial volume at visit 5, and the frequency of TV watching during leisure time. Alzheimer’s disease (AD) signature region includes volume of the parahippocampal, entorhinal, inferior parietal lobules, hippocampus, and precuneus.

# **Supplementary Table 8.** Interaction between frequency of TV watching and sex in regional brain volumes between the lowest (Never/Seldom) and the highest (Very often) frequency of TV watching. ARIC. N=1,712.

| **Brain region** | **Hemisphere** | **Beta (mm^3^)** | **P-value** | **FDR P-value** |
| --- | --- | --- | --- | --- |
| Amygdala | Left | -24.28 | 0.038 | 0.066 |
| Bankssts | Left | -41.41 | 0.064 | 0.103 |
| Caudal anterior cingulate | Left | -31.62 | 0.185 | 0.236 |
| Caudal middle frontal | Left | 15.08 | 0.756 | 0.805 |
| Caudate | Left | -65.91 | 0.030 | 0.054 |
| Cuneus | Left | -77.84 | 0.001 | 0.003 |
| Entorhinal | Left | -4.87 | 0.803 | 0.845 |
| Frontal pole | Left | -21.54 | 0.009 | 0.021 |
| Fusiform | Left | -60.95 | 0.359 | 0.423 |
| Hippocampus | Left | -34.19 | 0.194 | 0.245 |
| Inferior parietal | Left | -189.93 | 0.019 | 0.038 |
| Inferior temporal | Left | -242.68 | 0.001 | 0.005 |
| Insula | Left | -121.90 | 0.001 | 0.004 |
| Isthmus cingulate | Left | -96.12 | 0.000 | 0.000 |
| Lateral occipital | Left | -208.82 | 0.005 | 0.013 |
| Lateral orbitofrontal | Left | -59.64 | 0.092 | 0.138 |
| Lingual | Left | -30.99 | 0.584 | 0.643 |
| Medial orbitofrontal | Left | -45.71 | 0.122 | 0.167 |
| Middle temporal | Left | -149.32 | 0.028 | 0.053 |
| Pallidum | Left | -37.49 | 0.002 | 0.005 |
| Paracentral | Left | -52.32 | 0.047 | 0.078 |
| Parahippocampal | Left | -54.56 | 0.004 | 0.013 |
| Pars opercularis | Left | -108.10 | 0.005 | 0.013 |
| Pars orbitalis | Left | -45.85 | 0.003 | 0.008 |
| Pars triangularis | Left | -71.30 | 0.014 | 0.030 |
| Pericalcarine | Left | 0.72 | 0.971 | 0.981 |
| Postcentral | Left | -14.68 | 0.812 | 0.847 |
| Posterior cingulate | Left | -20.81 | 0.401 | 0.463 |
| Precentral | Left | -148.43 | 0.029 | 0.053 |
| Precuneus | Left | -118.28 | 0.016 | 0.034 |
| Putamen | Left | -182.55 | 0.000 | 0.000 |
| Rostral anterior cingulate | Left | -55.66 | 0.022 | 0.044 |
| Rostral middle frontal | Left | -280.57 | 0.000 | 0.002 |
| Superior frontal | Left | -506.28 | 0.000 | 0.000 |
| Superior parietal | Left | -275.91 | 0.000 | 0.002 |
| Superior temporal | Left | -159.69 | 0.009 | 0.021 |
| Supramarginal | Left | -199.51 | 0.003 | 0.009 |
| Temporal pole | Left | 0.08 | 0.997 | 0.997 |
| Thalamus proper | Left | -4.49 | 0.877 | 0.900 |
| Transverse temporal | Left | -15.35 | 0.181 | 0.233 |
| Amygdala | Right | -13.32 | 0.222 | 0.278 |
| Bankssts | Right | -12.59 | 0.540 | 0.600 |
| Caudal anterior cingulate | Right | -27.72 | 0.286 | 0.354 |
| Caudal middle frontal | Right | -155.12 | 0.001 | 0.005 |
| Caudate | Right | -46.85 | 0.104 | 0.150 |
| Cuneus | Right | -52.71 | 0.043 | 0.074 |
| Entorhinal | Right | -47.22 | 0.026 | 0.050 |
| Frontal pole | Right | -15.84 | 0.116 | 0.163 |
| Fusiform | Right | -54.53 | 0.398 | 0.463 |
| Hippocampus | Right | -89.76 | 0.001 | 0.003 |
| Inferior parietal | Right | -388.57 | 0.000 | 0.000 |
| Inferior temporal | Right | -302.42 | 0.000 | 0.001 |
| Insula | Right | -99.91 | 0.010 | 0.022 |
| Isthmus cingulate | Right | -68.61 | 0.000 | 0.002 |
| Lateral occipital | Right | -276.14 | 0.000 | 0.002 |
| Lateral orbitofrontal | Right | -1.21 | 0.972 | 0.981 |
| Lingual | Right | -31.33 | 0.537 | 0.600 |
| Medial orbitofrontal | Right | -45.94 | 0.113 | 0.161 |
| Middle temporal | Right | -175.95 | 0.008 | 0.019 |
| Pallidum | Right | -34.73 | 0.001 | 0.003 |
| Paracentral | Right | -63.68 | 0.032 | 0.057 |
| Parahippocampal | Right | -31.69 | 0.066 | 0.104 |
| Pars opercularis | Right | -49.17 | 0.138 | 0.185 |
| Pars orbitalis | Right | -70.94 | 0.000 | 0.002 |
| Pars triangularis | Right | -14.57 | 0.676 | 0.737 |
| Pericalcarine | Right | -36.73 | 0.093 | 0.138 |
| Postcentral | Right | 99.87 | 0.090 | 0.138 |
| Posterior cingulate | Right | -61.04 | 0.008 | 0.020 |
| Precentral | Right | -27.21 | 0.702 | 0.759 |
| Precuneus | Right | -171.80 | 0.001 | 0.003 |
| Putamen | Right | -170.76 | 0.000 | 0.000 |
| Rostral anterior cingulate | Right | -4.90 | 0.820 | 0.848 |
| Rostral middle frontal | Right | -337.21 | 0.000 | 0.000 |
| Superior frontal | Right | -481.07 | 0.000 | 0.000 |
| Superior parietal | Right | -214.93 | 0.006 | 0.015 |
| Superior temporal | Right | -138.92 | 0.017 | 0.034 |
| Supramarginal | Right | -187.81 | 0.005 | 0.013 |
| Temporal pole | Right | -95.55 | 0.000 | 0.000 |
| Thalamus proper | Right | -68.38 | 0.015 | 0.033 |
| Transverse temporal | Right | -2.70 | 0.758 | 0.805 |

Generalized linear models adjusted for gender, age (visit 5), race-center, education, occupation, BMI, diabetes, hypertension, visit 1, physical activity, estimated total intracranial volume at visit 5, and frequency of sitting during work. Bankssts, banks of the superior temporal sulcus.

# **Supplementary Table 9.** Interaction between frequency of sitting during work and sex in regional brain volumes between the lowest (Never/Seldom) and the highest (Always) frequency of sitting during work. ARIC. N=1,712.

| **Brain region** | **Hemisphere** | **Beta (mm^3^)** | **P-value** | **FDR P-value** |
| --- | --- | --- | --- | --- |
| Amygdala | Left | -6.76 | 0.397 | 0.619 |
| Bankssts | Left | -0.47 | 0.975 | 0.983 |
| Caudal anterior cingulate | Left | 57.87 | 0.000 | 0.017 |
| Caudal middle frontal | Left | -30.36 | 0.358 | 0.612 |
| Caudate | Left | -5.34 | 0.796 | 0.893 |
| Cuneus | Left | 2.91 | 0.852 | 0.912 |
| Entorhinal | Left | -45.94 | 0.001 | 0.017 |
| Frontal pole | Left | -1.30 | 0.817 | 0.893 |
| Fusiform | Left | -57.83 | 0.201 | 0.447 |
| Hippocampus | Left | -30.07 | 0.093 | 0.285 |
| Inferior parietal | Left | 48.20 | 0.381 | 0.612 |
| Inferior temporal | Left | 50.18 | 0.325 | 0.610 |
| Insula | Left | 36.14 | 0.147 | 0.367 |
| Isthmus cingulate | Left | 45.68 | 0.001 | 0.023 |
| Lateral occipital | Left | 143.21 | 0.004 | 0.038 |
| Lateral orbitofrontal | Left | 39.58 | 0.101 | 0.295 |
| Lingual | Left | -68.55 | 0.075 | 0.252 |
| Medial orbitofrontal | Left | 34.01 | 0.091 | 0.285 |
| Middle temporal | Left | 43.83 | 0.346 | 0.612 |
| Pallidum | Left | -6.98 | 0.388 | 0.612 |
| Paracentral | Left | 52.18 | 0.004 | 0.036 |
| Parahippocampal | Left | -35.15 | 0.007 | 0.044 |
| Pars opercularis | Left | 42.48 | 0.105 | 0.301 |
| Pars orbitalis | Left | 13.43 | 0.194 | 0.447 |
| Pars triangularis | Left | 11.74 | 0.552 | 0.744 |
| Pericalcarine | Left | -3.46 | 0.796 | 0.893 |
| Postcentral | Left | 26.03 | 0.535 | 0.730 |
| Posterior cingulate | Left | 56.65 | 0.001 | 0.019 |
| Precentral | Left | 67.50 | 0.144 | 0.367 |
| Precuneus | Left | -0.45 | 0.989 | 0.989 |
| Putamen | Left | 3.36 | 0.889 | 0.928 |
| Rostral anterior cingulate | Left | 48.75 | 0.003 | 0.035 |
| Rostral middle frontal | Left | 39.52 | 0.461 | 0.699 |
| Superior frontal | Left | -60.43 | 0.353 | 0.612 |
| Superior parietal | Left | 26.60 | 0.616 | 0.768 |
| Superior temporal | Left | 27.39 | 0.511 | 0.709 |
| Supramarginal | Left | 39.93 | 0.382 | 0.612 |
| Temporal pole | Left | 5.46 | 0.724 | 0.842 |
| Thalamus proper | Left | 25.48 | 0.199 | 0.447 |
| Transverse temporal | Left | -4.21 | 0.587 | 0.758 |
| Amygdala | Right | -18.15 | 0.015 | 0.075 |
| Bankssts | Right | -3.63 | 0.796 | 0.893 |
| Caudal anterior cingulate | Right | 19.60 | 0.268 | 0.554 |
| Caudal middle frontal | Right | -55.03 | 0.097 | 0.292 |
| Caudate | Right | -8.00 | 0.683 | 0.804 |
| Cuneus | Right | 15.73 | 0.376 | 0.612 |
| Entorhinal | Right | -34.45 | 0.017 | 0.079 |
| Frontal pole | Right | 19.33 | 0.005 | 0.039 |
| Fusiform | Right | -19.33 | 0.661 | 0.793 |
| Hippocampus | Right | -45.84 | 0.010 | 0.060 |
| Inferior parietal | Right | -34.63 | 0.582 | 0.758 |
| Inferior temporal | Right | 46.51 | 0.373 | 0.612 |
| Insula | Right | -14.78 | 0.573 | 0.758 |
| Isthmus cingulate | Right | -9.44 | 0.480 | 0.709 |
| Lateral occipital | Right | 112.58 | 0.028 | 0.119 |
| Lateral orbitofrontal | Right | 94.86 | 0.000 | 0.008 |
| Lingual | Right | -17.89 | 0.604 | 0.763 |
| Medial orbitofrontal | Right | 46.85 | 0.018 | 0.079 |
| Middle temporal | Right | 30.99 | 0.491 | 0.709 |
| Pallidum | Right | 3.64 | 0.595 | 0.759 |
| Paracentral | Right | 31.42 | 0.119 | 0.326 |
| Parahippocampal | Right | -23.77 | 0.043 | 0.171 |
| Pars opercularis | Right | 14.86 | 0.512 | 0.709 |
| Pars orbitalis | Right | 24.02 | 0.071 | 0.248 |
| Pars triangularis | Right | -3.10 | 0.896 | 0.928 |
| Pericalcarine | Right | 6.28 | 0.673 | 0.799 |
| Postcentral | Right | 29.24 | 0.466 | 0.699 |
| Posterior cingulate | Right | 29.83 | 0.056 | 0.212 |
| Precentral | Right | 116.04 | 0.016 | 0.079 |
| Precuneus | Right | 95.83 | 0.006 | 0.044 |
| Putamen | Right | -25.99 | 0.282 | 0.566 |
| Rostral anterior cingulate | Right | -1.27 | 0.931 | 0.946 |
| Rostral middle frontal | Right | 62.23 | 0.266 | 0.554 |
| Superior frontal | Right | 195.50 | 0.003 | 0.035 |
| Superior parietal | Right | 56.91 | 0.283 | 0.566 |
| Superior temporal | Right | 7.59 | 0.848 | 0.912 |
| Supramarginal | Right | 30.45 | 0.502 | 0.709 |
| Temporal pole | Right | -36.26 | 0.009 | 0.058 |
| Thalamus proper | Right | 17.35 | 0.366 | 0.612 |
| Transverse temporal | Right | -3.33 | 0.577 | 0.758 |

Generalized linear models adjusted for gender, age (visit 5), race-center, education, occupation, BMI, diabetes, hypertension, visit 1, physical activity, estimated total intracranial volume at visit 5, and frequency of TV. Bankssts, banks of the superior temporal sulcus.

# **Supplementary Table 10.** Sensitivity analysis on the association between sedentary behavior and cortical, subcortical, and white matter hyperintensity volumes, excluding participants with cerebrovascular disease-related MCI. N=1,612. ARIC study.

|  | TV watching | | | | Sitting during work | | | | | |
| --- | --- | --- | --- | --- | --- | --- | --- | --- | --- | --- |
| Brain region | Never/  seldom | Sometimes | Often | Very often | Never/  seldom | Sometimes | Often | Always | Do not work |  |
| *AD signature* | 1.00 | 0.03 (-0.05, 0.11) | -0.02 (-0.11, 0.07) | **-0.19 (-0.35, -0.03)** | 1.00 | 0.05 (-0.04, 0.14) | 0.08 (-0.02, 0.17) | **0.15 (0.04, 0.26)** | 0 (-0.13, 0.14) |  |
| *Frontal CV* | 1.00 | -0.04 (-0.11, 0.04) | -0.09 (-0.18, -0.01) | -0.18 (-0.33, -0.03) | 1.00 | **0.11 (0.02, 0.19)** | **0.18 (0.09, 0.27)** | **0.13 (0.03, 0.23)** | 0.03 (-0.09, 0.16) |  |
| *Temporal CV* | 1.00 | -0.01 (-0.09, 0.07) | -0.03 (-0.12, 0.06) | -0.11 (-0.27, 0.05) | 1.00 | **0.15 (0.06, 0.24)** | **0.14 (0.05, 0.23)** | 0.12 (0.01, 0.23) | 0.02 (-0.12, 0.15) |  |
| *Occipital CV* | 1.00 | -0.09 (-0.18, 0) | 0 (-0.1, 0.1) | -0.16 (-0.34, 0.02) | 1.00 | **0.12 (0.02, 0.22)** | 0.1 (0, 0.21) | **0.23 (0.1, 0.35)** | 0.15 (0, 0.3) |  |
| *Parietal CV* | 1.00 | 0.04 (-0.03, 0.12) | -0.03 (-0.12, 0.05) | -0.11 (-0.25, 0.04) | 1.00 | **0.11 (0.03, 0.2)** | **0.11 (0.03, 0.2)** | **0.2 (0.09, 0.3)** | 0.09 (-0.04, 0.21) |  |
| *Deep gray CV* | 1.00 | 0 (-0.1, 0.09) | -0.02 (-0.13, 0.09) | -0.03 (-0.22, 0.16) | 1.00 | 0 (-0.11, 0.11) | 0.01 (-0.1, 0.12) | 0.1 (-0.03, 0.24) | 0.01 (-0.15, 0.18) |  |
| *WMH volume* | 1.00 | 0.04 (-0.08, 0.16) | -0.05 (-0.18, 0.09) | **0.42 (0.19, 0.66)** | 1.00 | -0.01 (-0.15, 0.13) | 0.11 (-0.03, 0.24) | -0.12 (-0.29, 0.04) | 0.02 (-0.18, 0.22) |  |

Values are beta coefficients and respective 95% confidence intervals extracted from multivariate generalized linear models.

**Bold values indicate false discovery rate-adjusted p<0.05**

Generalized linear models adjusted for gender, race-center, education, BMI, diabetes, hypertension, and physical activity at visit 1, age and estimated total intracranial volume at visit 5, and mutually adjusted for frequency of TV and sitting during work.

# **Supplementary Table 11.** Sensitivity analysis on the association between sedentary behavior, excluding participants with mild cognitive impairment. N=1,036. ARIC study.

|  | TV watching | | | | Sitting during work | | | | | |
| --- | --- | --- | --- | --- | --- | --- | --- | --- | --- | --- |
| Brain region | Never/  seldom | Sometimes | Often | Very often | Never/  seldom | Sometimes | Often | Always | Do not work |  |
| *AD signature* | 1.00 | 0.05 (-0.05, 0.15) | 0 (-0.12, 0.11) | -0.21 (-0.4, -0.02) | 1.00 | 0.08 (-0.04, 0.19) | 0.09 (-0.03, 0.2) | **0.16 (0.03, 0.3)** | 0.03 (-0.14, 0.2) |  |
| *Frontal CV* | 1.00 | -0.05 (-0.15, 0.04) | -0.09 (-0.2, 0.02) | -0.13 (-0.32, 0.05) | 1.00 | 0.13 (0.02, 0.24) | **0.23 (0.12, 0.34)** | **0.17 (0.04, 0.3)** | 0.07 (-0.1, 0.23) |  |
| *Temporal CV* | 1.00 | 0.04 (-0.06, 0.13) | 0 (-0.11, 0.11) | -0.08 (-0.27, 0.11) | 1.00 | **0.2 (0.09, 0.31)** | **0.15 (0.04, 0.26)** | 0.15 (0.02, 0.28) | 0.05 (-0.11, 0.22) |  |
| *Occipital CV* | 1.00 | -0.08 (-0.19, 0.04) | 0.05 (-0.08, 0.18) | -0.17 (-0.39, 0.04) | 1.00 | 0.13 (0, 0.26) | 0.13 (0, 0.26) | **0.21 (0.06, 0.36)** | 0.16 (-0.03, 0.35) |  |
| *Parietal CV* | 1.00 | 0.05 (-0.04, 0.15) | -0.03 (-0.14, 0.08) | -0.12 (-0.3, 0.07) | 1.00 | **0.14 (0.03, 0.25)** | **0.14 (0.03, 0.25)** | **0.22 (0.09, 0.35)** | 0.16 (0, 0.32) |  |
| *Deep gray CV* | 1.00 | 0.05 (-0.08, 0.17) | 0.03 (-0.11, 0.17) | 0.05 (-0.18, 0.29) | 1.00 | 0.05 (-0.09, 0.19) | 0.02 (-0.12, 0.16) | 0.1 (-0.06, 0.26) | 0.02 (-0.18, 0.23) |  |
| *WMH volume* | 1.00 | 0.13 (-0.02, 0.27) | 0.08 (-0.09, 0.24) | **0.52 (0.24, 0.8)** | 1.00 | -0.15 (-0.32, 0.01) | 0.03 (-0.14, 0.19) | **-0.27 (-0.46, -0.08)** | -0.04 (-0.28, 0.2) |  |

Values are beta coefficients and respective 95% confidence intervals extracted from multivariate generalized linear models.

**Bold values indicate false discovery rate-adjusted p<0.05**

Generalized linear models adjusted for gender, race-center, education, BMI, diabetes, hypertension, and physical activity at visit 1, age and estimated total intracranial volume at visit 5, and mutually adjusted for frequency of TV and sitting during work.

# **Supplementary Table 12.** Sensitivity analysis on the association between sedentary behavior and cortical, subcortical, and white matter hyperintensity volumes, additionally adjusting for *APOE* ɛ4 genotype. N=822. ARIC study.

|  | TV watching | | | | Sitting during work | | | | | |
| --- | --- | --- | --- | --- | --- | --- | --- | --- | --- | --- |
| Brain region | Never/  seldom | Sometimes | Often | Very often | Never/  seldom | Sometimes | Often | Always | Do not work |  |
| *AD signature* | 1.00 | 0.12 (0.01, 0.23) | 0.05 (-0.08, 0.17) | -0.24 (-0.51, 0.03) | 1.00 | 0.07 (-0.07, 0.21) | 0.06 (-0.08, 0.19) | 0.11 (-0.06, 0.28) | -0.07 (-0.27, 0.14) |  |
| *Frontal CV* | 1.00 | -0.04 (-0.14, 0.06) | -0.02 (-0.14, 0.1) | **-0.37 (-0.62, -0.11)** | 1.00 | 0.03 (-0.11, 0.16) | 0.1 (-0.03, 0.23) | 0.07 (-0.09, 0.23) | -0.06 (-0.25, 0.13) |  |
| *Temporal CV* | 1.00 | 0.1 (-0.01, 0.21) | 0.04 (-0.09, 0.16) | -0.02 (-0.29, 0.24) | 1.00 | 0.14 (0, 0.28) | 0.12 (-0.01, 0.26) | 0.13 (-0.03, 0.3) | 0.02 (-0.18, 0.22) |  |
| *Occipital CV* | 1.00 | -0.11 (-0.24, 0.02) | -0.01 (-0.15, 0.14) | **-0.47 (-0.79, -0.15)** | 1.00 | 0.09 (-0.08, 0.26) | 0.03 (-0.13, 0.2) | 0.14 (-0.06, 0.34) | 0.06 (-0.18, 0.29) |  |
| *Parietal CV* | 1.00 | 0.12 (0.02, 0.23) | 0 (-0.13, 0.12) | -0.08 (-0.33, 0.18) | 1.00 | 0.14 (0.01, 0.28) | 0.11 (-0.02, 0.24) | 0.19 (0.03, 0.36) | -0.02 (-0.22, 0.17) |  |
| *Deep gray CV* | 1.00 | -0.06 (-0.2, 0.07) | -0.03 (-0.18, 0.12) | -0.21 (-0.54, 0.11) | 1.00 | -0.09 (-0.26, 0.08) | -0.1 (-0.27, 0.06) | -0.14 (-0.34, 0.07) | -0.21 (-0.45, 0.04) |  |
| *WMH volume* | 1.00 | -0.03 (-0.18, 0.13) | 0.09 (-0.09, 0.27) | **0.62 (0.23, 1.00)** | 1.00 | -0.14 (-0.34, 0.06) | -0.01 (-0.21, 0.18) | **-0.30 (-0.54, -0.06)** | 0.11 (-0.18, 0.39) |  |

Values are beta coefficients and respective 95% confidence intervals extracted from multivariate generalized linear models.

**Bold values indicate false discovery rate-adjusted p<0.05**

Generalized linear models adjusted for *APOE* ɛ4 genotype, gender, race-center, education, BMI, diabetes, hypertension, and physical activity at visit 1, age and estimated total intracranial volume at visit 5, and mutually adjusted for frequency of TV and sitting during work.

# **Supplementary Table 13.** Sensitivity analysis on the association between sedentary behavior and cortical, subcortical, and white matter hyperintensity volumes accounting for inverse probability attrition weighting. N=1,712. ARIC study.

|  | TV watching | | | | Sitting during work | | | | | |
| --- | --- | --- | --- | --- | --- | --- | --- | --- | --- | --- |
| Brain region | Never/  seldom | Sometimes | Often | Very often | Never/  seldom | Sometimes | Often | Always | Do not work |  |
| *AD signature* | 1.00 | 0.01 (-0.08, 0.09) | -0.03 (-0.12, 0.06) | -0.15 (-0.29, -0.02) | 1.00 | 0.02 (-0.07, 0.1) | 0.06 (-0.03, 0.15) | **0.14 (0.03, 0.24)** | 0 (-0.12, 0.11) |  |
| *Frontal CV* | 1.00 | -0.04 (-0.12, 0.04) | -0.09 (-0.18, 0) | **-0.2 (-0.33, -0.07)** | 1.00 | **0.12 (0.04, 0.2)** | **0.19 (0.11, 0.28)** | **0.12 (0.02, 0.23)** | 0.02 (-0.09, 0.14) |  |
| *Temporal CV* | 1.00 | -0.01 (-0.1, 0.07) | -0.02 (-0.11, 0.07) | -0.04 (-0.17, 0.1) | 1.00 | **0.08 (-0.01, 0.16)** | **0.12 (0.03, 0.21)** | 0.11 (0, 0.21) | 0.03 (-0.08, 0.15) |  |
| *Occipital CV* | 1.00 | -0.06 (-0.15, 0.03) | 0.04 (-0.06, 0.14) | -0.13 (-0.28, 0.02) | 1.00 | 0.11 (0.02, 0.21) | 0.11 (0.01, 0.21) | **0.29 (0.17, 0.41)** | 0.14 (0.01, 0.28) |  |
| *Parietal CV* | 1.00 | 0.04 (-0.04, 0.12) | -0.01 (-0.1, 0.07) | -0.07 (-0.2, 0.06) | 1.00 | 0.06 (-0.03, 0.14) | **0.11 (0.02, 0.19)** | **0.16 (0.06, 0.26)** | 0.05 (-0.06, 0.17) |  |
| *Deep gray CV* | 1.00 | 0.02 (-0.09, 0.12) | 0.03 (-0.08, 0.15) | -0.01 (-0.18, 0.16) | 1.00 | -0.12 (-0.23, -0.01) | -0.02 (-0.13, 0.09) | 0.08 (-0.06, 0.21) | -0.1 (-0.25, 0.05) |  |
| *WMH volume* | 1.00 | 0.09 (-0.02, 0.21) | 0.06 (-0.07, 0.19) | **0.49 (0.29, 0.68)** | 1.00 | -0.13 (-0.26, -0.01) | 0.04 (-0.09, 0.17) | **-0.24 (-0.4, -0.09)** | 0.04 (-0.13, 0.21) |  |

Values are beta coefficients and respective 95% confidence intervals extracted from multivariate generalized linear models.

**Bold values indicate false discovery rate-adjusted p<0.05**

Generalized linear models adjusted for gender, race-center, education, BMI, diabetes, hypertension, and physical activity at visit 1, age and estimated total intracranial volume at visit 5, and mutually adjusted for frequency of TV and sitting during work.

# **Supplementary Table 14.** Sensitivity analysis on the association between time-fixed sedentary behavior and cortical, subcortical, and white matter hyperintensity volumes adjusting for time-varying covariates. N=1,712. ARIC study.

|  | TV watching | | | | Sitting during work | | | | | |
| --- | --- | --- | --- | --- | --- | --- | --- | --- | --- | --- |
| Brain region | Never/  seldom | Sometimes | Often | Very often | Never/  seldom | Sometimes | Often | Always | Do not work |  |
| *AD signature* | 1.00 | 0.02 (-0.06, 0.1) | -0.03 (-0.12, 0.05) | **-0.21 (-0.36, -0.06)** | 1.00 | 0.03 (-0.06, 0.12) | 0.07 (-0.01, 0.16) | 0.07 (-0.04, 0.17) | -0.02 (-0.15, 0.11) |  |
| *Frontal CV* | 1.00 | -0.05 (-0.12, 0.02) | **-0.1 (-0.18, -0.01)** | **-0.23 (-0.38, -0.09)** | 1.00 | **0.1 (0.02, 0.18)** | **0.18 (0.09, 0.26)** | 0.12 (0.01, 0.22) | 0.01 (-0.11, 0.13) |  |
| *Temporal CV* | 1.00 | -0.02 (-0.1, 0.06) | -0.04 (-0.13, 0.04) | -0.13 (-0.27, 0.02) | 1.00 | **0.14 (0.05, 0.22)** | **0.14 (0.06, 0.23)** | 0.07 (-0.03, 0.18) | 0.01 (-0.11, 0.14) |  |
| *Occipital CV* | 1.00 | -0.08 (-0.17, 0.01) | 0 (-0.1, 0.1) | -0.17 (-0.34, 0) | 1.00 | **0.12 (0.02, 0.22)** | 0.12 (0.02, 0.22) | **0.2 (0.08, 0.33)** | 0.13 (-0.02, 0.27) |  |
| *Parietal CV* | 1.00 | 0.04 (-0.03, 0.11) | -0.04 (-0.12, 0.04) | -0.12 (-0.26, 0.02) | 1.00 | 0.09 (0.01, 0.17) | **0.11 (0.03, 0.19)** | **0.14 (0.04, 0.24)** | 0.04 (-0.08, 0.16) |  |
| *Deep gray CV* | 1.00 | 0 (-0.09, 0.1) | -0.03 (-0.13, 0.08) | -0.08 (-0.26, 0.11) | 1.00 | -0.03 (-0.14, 0.08) | 0 (-0.11, 0.1) | 0.03 (-0.1, 0.16) | -0.04 (-0.2, 0.12) |  |
| *WMH volume* | 1.00 | 0.08 (-0.03, 0.2) | 0.07 (-0.06, 0.19) | **0.43 (0.21, 0.64)** | 1.00 | -0.13 (-0.25, 0) | 0.01 (-0.12, 0.13) | **-0.23 (-0.38, -0.07)** | 0.01 (-0.17, 0.19) |  |

Values are beta coefficients and respective 95% confidence intervals extracted from multivariate generalized linear models.

**Bold values indicate false discovery rate-adjusted p<0.05**

Generalized linear models adjusted for gender, race-center, and education at visit 1, age and estimated total intracranial volume at visit 5, smoking, alcohol consumption, BMI, diabetes, hypertension, and physical activity across follow-up, and mutually adjusted for frequency of TV and sitting during work.

# **Supplementary Table 15.** Sensitivity analysis on the association between time-varying sedentary behavior and cortical, subcortical, and white matter hyperintensity volumes adjusting for time-varying covariates. N=1,712. ARIC study.

|  | TV watching | | | | Sitting during work | | | | | |
| --- | --- | --- | --- | --- | --- | --- | --- | --- | --- | --- |
| Brain region | Never/  seldom | Sometimes | Often | Very often | Never/  seldom | Sometimes | Often | Always | Do not work |  |
| *AD signature* | 1.00 | 0.02 (-0.06, 0.1) | -0.03 (-0.12, 0.05) | **-0.21 (-0.36, -0.06)** | 1.00 | 0.03 (-0.06, 0.12) | 0.07 (-0.01, 0.16) | 0.07 (-0.04, 0.17) | -0.02 (-0.15, 0.11) |  |
| *Frontal CV* | 1.00 | -0.05 (-0.12, 0.02) | **-0.1 (-0.18, -0.01)** | **-0.23 (-0.38, -0.09)** | 1.00 | **0.1 (0.02, 0.18)** | **0.18 (0.09, 0.26)** | **0.12 (0.02, 0.22)** | 0.01 (-0.11, 0.13) |  |
| *Temporal CV* | 1.00 | -0.02 (-0.1, 0.06) | -0.04 (-0.13, 0.04) | -0.13 (-0.27, 0.02) | 1.00 | **0.14 (0.05, 0.22)** | **0.14 (0.06, 0.23)** | 0.07 (-0.03, 0.18) | 0.01 (-0.11, 0.14) |  |
| *Occipital CV* | 1.00 | -0.08 (-0.17, 0.01) | 0 (-0.1, 0.1) | -0.16 (-0.33, 0.01) | 1.00 | **0.12 (0.02, 0.22)** | **0.12 (0.02, 0.22)** | **0.21 (0.09, 0.33)** | 0.13 (-0.01, 0.28) |  |
| *Parietal CV* | 1.00 | 0.04 (-0.03, 0.11) | -0.04 (-0.12, 0.04) | -0.12 (-0.26, 0.02) | 1.00 | 0.09 (0.01, 0.17) | **0.11 (0.03, 0.19)** | **0.14 (0.04, 0.24)** | 0.04 (-0.08, 0.16) |  |
| *Deep gray CV* | 1.00 | 0 (-0.09, 0.1) | -0.03 (-0.13, 0.08) | -0.08 (-0.26, 0.1) | 1.00 | -0.03 (-0.14, 0.08) | 0 (-0.11, 0.1) | 0.03 (-0.1, 0.16) | -0.04 (-0.2, 0.12) |  |
| *WMH volume* | 1.00 | 0.08 (-0.03, 0.19) | 0.06 (-0.06, 0.19) | **0.42 (0.21, 0.64)** | 1.00 | -0.13 (-0.26, -0.01) | 0 (-0.12, 0.13) | **-0.24 (-0.39, -0.08)** | 0.01 (-0.18, 0.19) |  |

Values are beta coefficients and respective 95% confidence intervals extracted from multivariate generalized linear models.

**Bold values indicate false discovery rate-adjusted p<0.05**

Generalized linear models adjusted for gender, race-center, and education at visit 1, age and estimated total intracranial volume at visit 5, smoking, alcohol consumption, BMI, diabetes, hypertension, and physical activity across follow-up, and mutually adjusted for frequency of TV and sitting during work.


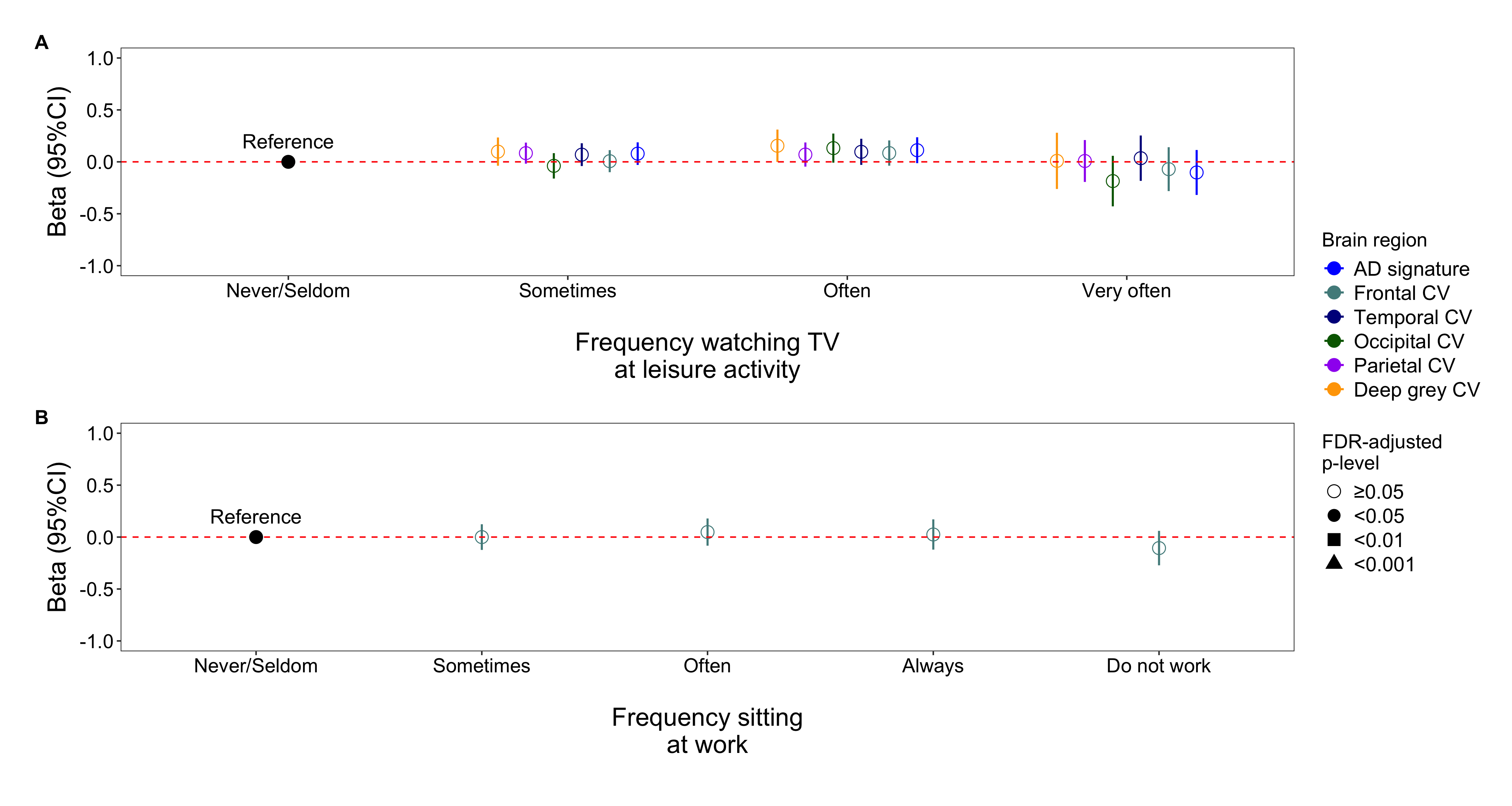


# **Supplementary Figure 1.** Association between brain structure and frequency of watching TV in leisure time (A) and sitting during work (B) in females. N=981. ARIC study.

Generalized linear models included race-center, occupational status, schooling, body mass index, diabetes, hypertension, physical activity at visit 1, age and intracranial volume at visit 5, and the frequency of TV watching and sitting during work. Alzheimer’s disease (AD) signature region includes volume of the parahippocampal, entorhinal, inferior parietal lobules, hippocampus, and precuneus.

AD: Alzheimer disease; CV: cortical volume; WMH: white matter hyperintensity; CI = confidence interval.


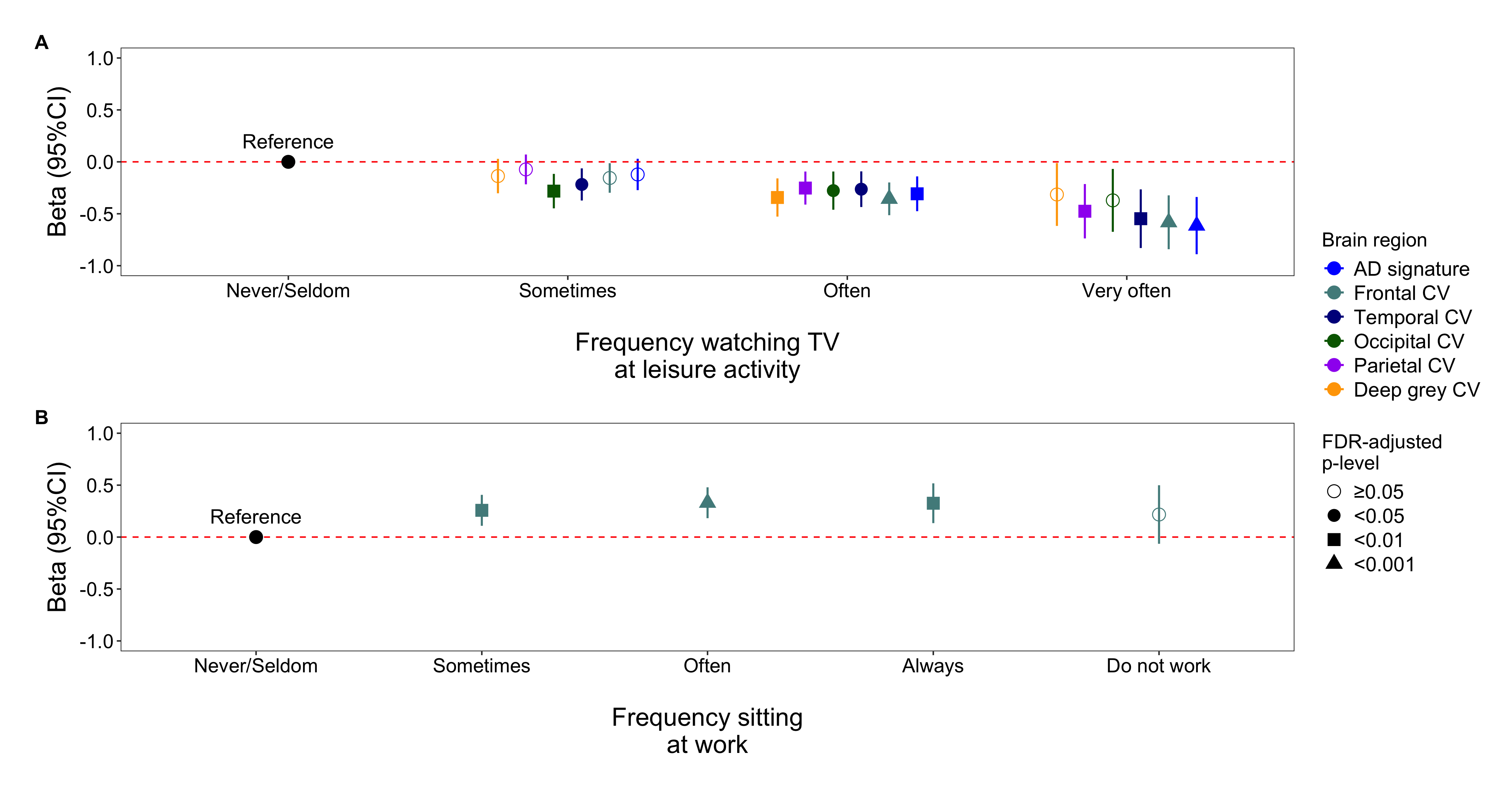


# **Supplementary Figure 2.** Association between brain structure and the frequency of watching TV in leisure time (A) and sitting during work (B) in males. N=731. ARIC study.

Generalized linear models included race-center, occupational status, schooling, body mass index, diabetes, hypertension, physical activity at visit 1, age and intracranial volume at visit 5, and the frequency of TV watching and sitting during work. Alzheimer’s disease (AD) signature region includes volume of the parahippocampal, entorhinal, inferior parietal lobules, hippocampus, and precuneus.

AD: Alzheimer disease; CV: cortical volume; WMH: white matter hyperintensity; CI = confidence interval.


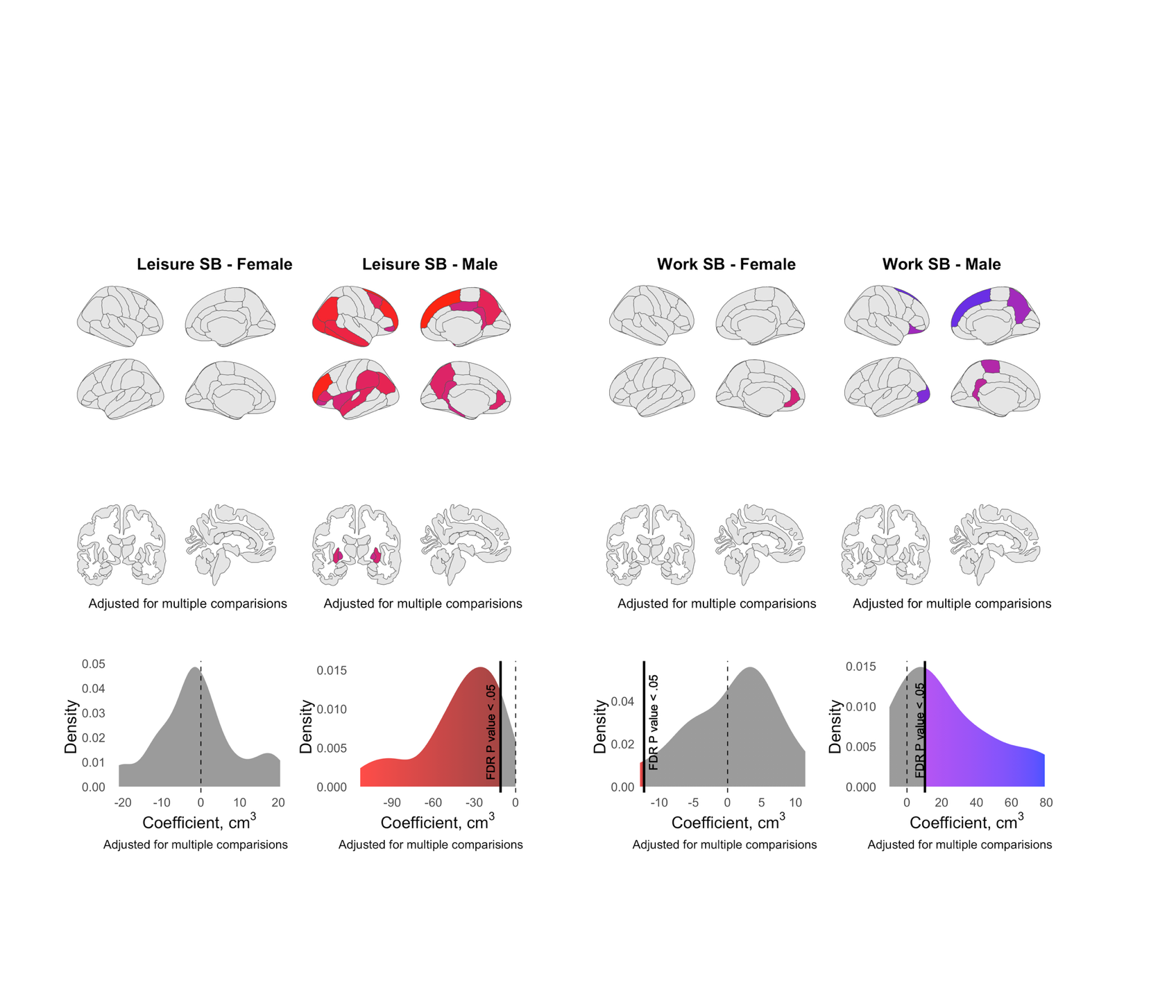


# **Supplementary Figure 3.** Association between context-specific sedentary behavior and brain structure according to sex. N=1,712. ARIC study.

Red and blue areas indicate a lower and greater cortical volume, respectively, in participants who reported the highest frequency on sedentary behavior in each context (TV watching: very often; sitting during work: always) compared to the least sedentary participants (i.e., “Never/seldom”). Generalized linear models adjusted for race-center, schooling, occupation, body mass index, diabetes, hypertension, and physical activity at visit 1, age and total intracranial volume at visit 5, and mutually adjusted for frequency of TV and sitting during work. The figure illustrates the brain regions that remained associated with high sedentary behavior (Panel A: very often TV watching; Panel B: always sitting at work) after controlling for multiple comparisons using the false discovery rate (FDR) procedure.
